# Coping with stress: How bacteria fine-tune protein synthesis and protein transport

**DOI:** 10.1016/j.jbc.2023.105163

**Published:** 2023-08-14

**Authors:** Robert Njenga, Julian Boele, Yavuz Öztürk, Hans-Georg Koch

**Affiliations:** 1 Faculty of Medicine, Institute for Biochemistry and Molecular Biology, ZBMZ, Albert-Ludwigs University Freiburg, Freiburg, Germany; 2 Faculty of Biology, Albert-Ludwigs University Freiburg, Freiburg, Germany

**Keywords:** alarmones, *Escherichia coli*, protein targeting, ribosome, RNA transport, signal recognition particle, stress response, stringent response

## Abstract

Maintaining a functional proteome under different environmental conditions is challenging for every organism, in particular for unicellular organisms, such as bacteria. In order to cope with changing environments and stress conditions, bacteria depend on strictly coordinated proteostasis networks that control protein production, folding, trafficking, and degradation. Regulation of ribosome biogenesis and protein synthesis are cornerstones of this cellular adaptation in all domains of life, which is rationalized by the high energy demand of both processes and the increased resistance of translationally silent cells against internal or external poisons. Reduced protein synthesis ultimately also reduces the substrate load for protein transport systems, which are required for maintaining the periplasmic, inner, and outer membrane subproteomes. Consequences of impaired protein transport have been analyzed in several studies and generally induce a multifaceted response that includes the upregulation of chaperones and proteases and the simultaneous downregulation of protein synthesis. In contrast, generally less is known on how bacteria adjust the protein targeting and transport machineries to reduced protein synthesis, *e.g.*, when cells encounter stress conditions or face nutrient deprivation. In the current review, which is mainly focused on studies using *Escherichia coli* as a model organism, we summarize basic concepts on how ribosome biogenesis and activity are regulated under stress conditions. In addition, we highlight some recent developments on how stress conditions directly impair protein targeting to the bacterial membrane. Finally, we describe mechanisms that allow bacteria to maintain the transport of stress-responsive proteins under conditions when the canonical protein targeting pathways are impaired.

The dynamic regulation of a balanced and compartmentalized proteome is essential for every organism and depends on a coordinated network of molecular machineries that control all aspects of the life cycle of proteins. This includes protein synthesis at the ribosome, co- or posttranslational protein folding, co- or posttranslational protein transport, and finally protein degradation (1, 2, 3, 4, 5, 6, 7, 8) (Fig. 1). Ribosomes serve as major checkpoints of this proteostasis network in pro- and eukaryotes and are crucial targets for stress-induced adaptation to nonfavorable conditions (9, 10, 11). This is explained by their cellular abundance, the high-energy demand of their biogenesis, and the costs of protein synthesis (11). In response to stress conditions or nutrient depletion, bacterial cells reduce ribosome biogenesis, modify ribosomes and components of the translational machinery, and regulate translation initiation and elongation for adapting protein synthesis to changing conditions (Fig. 1). Bacteria also silence ribosomes for protecting them against stress-induced damage (12, 13, 14, 15, 16, 17, 18, 19, 20). This adaptation is complemented by increased production of chaperones and proteases, which support correct folding under suboptimal conditions and degrade aggregated or damaged proteins (3, 21, 22). A similar response is also initiated when protein transport across membranes is impaired or saturated due to the limiting number of targeting factors and protein transport channels (6, 23, 24, 25, 26). Enhanced chaperone and protease production reduces the cytosolic aggregation of proteins that cannot be transported or induces their degradation (26, 27, 28, 29). Simultaneously, the membrane-bound protease FtsH clears jammed protein transport channels (30, 31) and the membrane surface is enlarged as a result of increased phospholipid biosynthesis (32, 33, 34). Finally, protein synthesis is downregulated and ribosomal proteins are sequestered in inactive aggregates (35, 36, 37). The upregulation of chaperones and proteases when protein targeting is impaired is mainly induced *via* the stress-responsive RNA polymerase (RNAP) subunit RpoH (σ^32^) (38, 39). For executing its stress-responsive function, σ^32^ needs to out-compete the housekeeping σ^70^ subunit, which requires the presence of the hyperphosphorylated guanine nucleotides ppGpp and pppGpp, collectively called alarmones (40, 41) (Fig. 1). Nucleotide second messengers such as (p)ppGpp act as critical signaling molecules during stress conditions and redirect the cellular metabolism toward the synthesis of stress resistance factors, while rapidly inhibiting the synthesis of ribosomal proteins or ribosomal RNA (rRNA) (11, 42, 43). Considering that approximately one-third of the entire *Escherichia coli* proteome (38, 44, 45) execute their function outside of the cytosol, the downregulation of protein synthesis is critical for preventing cytosolic protein aggregation when protein transport is impaired. On the other hand, it is largely unknown how the production and activities of the protein transport machineries are adapted to reduced protein synthesis, *e.g.*, when cells enter stationary phase or encounter nutrient starvation. Intriguingly, a recent study has demonstrated that (p)ppGpp accumulation not only reduces protein synthesis but also shuts down the essential signal recognition particle (SRP)-dependent protein targeting pathway in bacteria (46).

**Figure 1:**
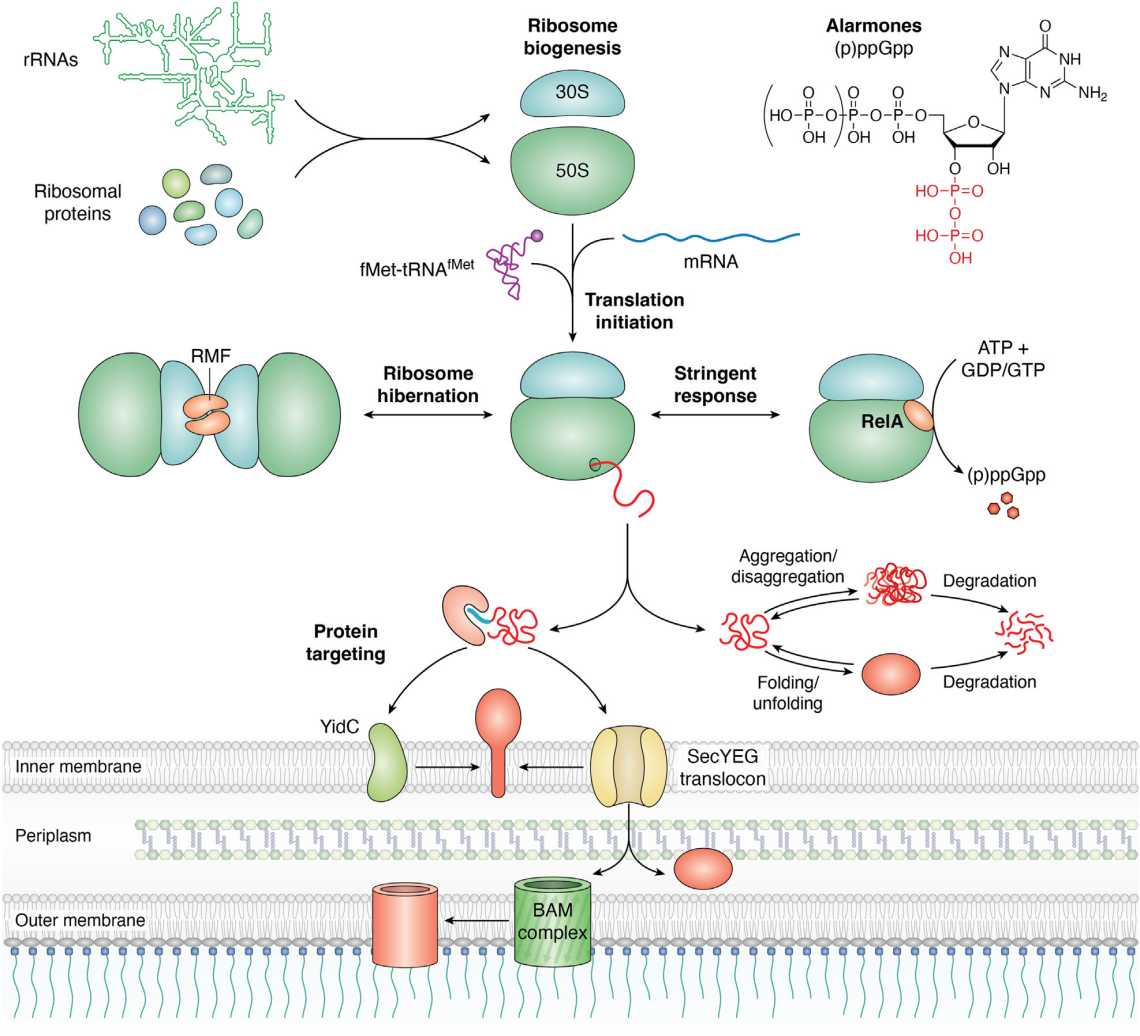
**General overview of ribosome biogenesis, ribosome hibernation, and the proteostasis network in *Escherichia coli*.** Ribosome biogenesis in *E. coli* requires the coordinated synthesis and association of the three distinct rRNAs (23S rRNA, 5S rRNA, and16S rRNA) with 54 ribosomal proteins (21 proteins for the 30S subunit and 33 proteins for the 50S subunit). Translation initiation is induced upon binding of the mRNA to the 30S ribosomal subunit and the subsequent recruitment of the 50S ribosomal subunit to form the translating 70S ribosome. The proteostasis network includes chaperones and proteases that assist protein folding and degrade misfolded, damaged, or aggregated proteins. Protein targeting factors are also part of this network and recognize N-terminal signal sequences (*blue*). They then deliver their cargo either co- or posttranslationally to the SecYEG translocon or to the YidC insertase, which constitute the main protein transport sites in *E. coli*. Most outer membrane proteins are inserted into the outer membrane by the BAM complex after their passage through the SecYEG channel. Under stress conditions, ribosome biogenesis is drastically reduced, primarily *via* a reduced transcription of the rRNA genes. In addition, ribosomes are inactivated by a process called ribosome hibernation. Furthermore, nutrient starvation induces the stringent response, which activates the ribosome-associated protein RelA and leads to the production of the hyperphosphorylated guanine nucleotides ppGpp and pppGpp, collectively called alarmones. (p)ppGpp influence all of the processes mentioned above.

In the first part of this review, we summarize the different strategies that bacteria use to regulate ribosome biogenesis and activity in response to stress conditions and how they engage different proteins for silencing ribosomes. The second part briefly highlights general concepts of Sec-dependent protein transport and then describes how the protein transport machinery responds to stress conditions and how this enables bacteria to coordinate protein transport with reduced protein synthesis.

## Ribosome assembly and protein synthesis under stress conditions

**Figure 2:**
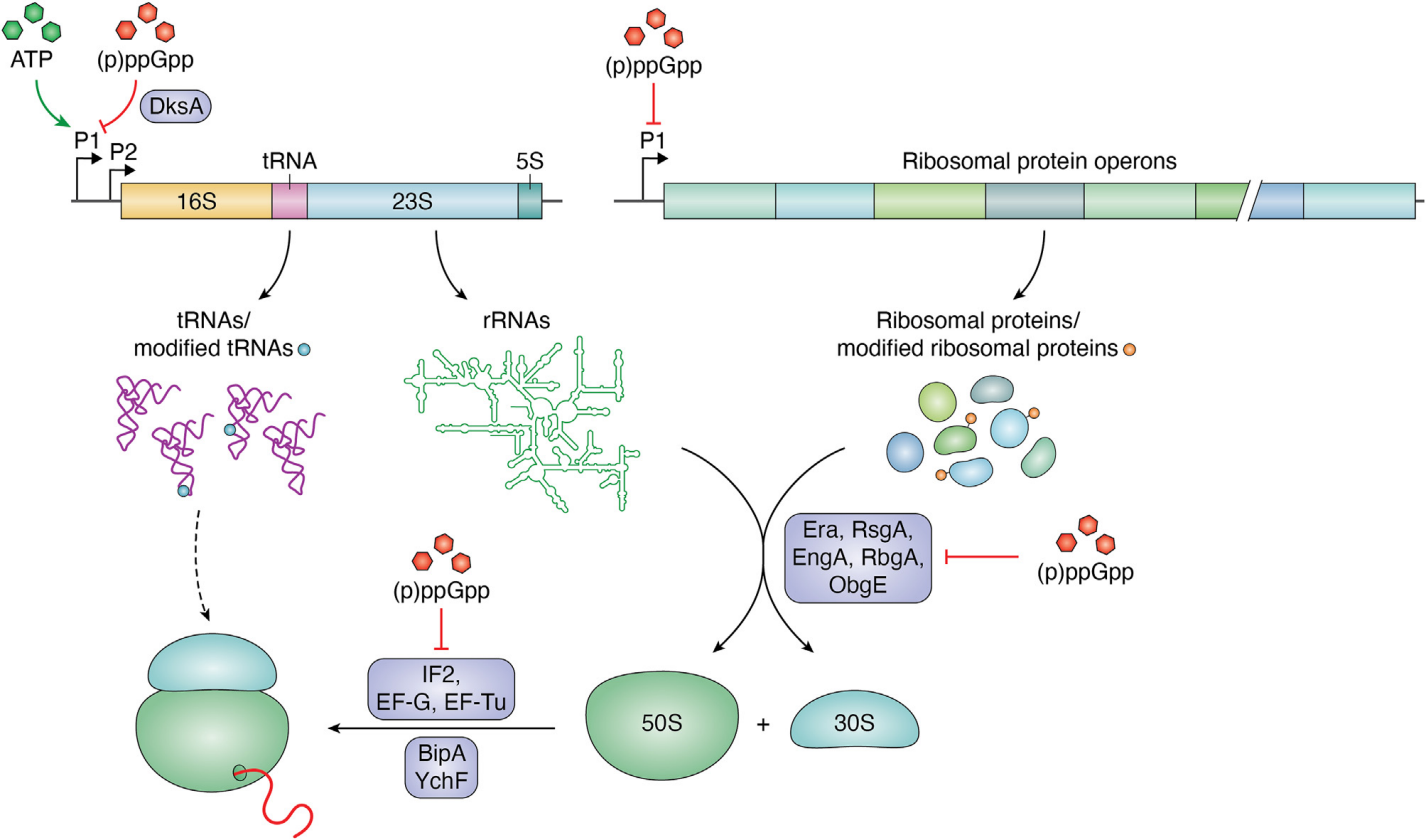
**Regulation of ribosome biogenesis and function in response to ATP and (p)ppGpp levels.** The transcription of the *rRNA* operons from the P1 promoter requires high ATP concentrations, and transcription is prevented by the (p)ppGpp-bound transcription factor DksA. In addition, (p)ppGpp prevent the transcription of the ribosomal protein operons. Modified ribosomal proteins and modified tRNAs refer to stress-induced modifications, such as phosphorylation or methylation. (p)ppGpp also compete with GTP for binding to GTP-dependent ribosome assembly factors and initiation and elongation factors, which leads to reduced ribosome assembly and reduced translation when (p)ppGpp accumulate. BipA and YchF refer to stress-responsive ribosome-interacting proteins that have been implicated in the selective translation of stress response proteins.

A single *E. coli* cell can contain up to 70,000 ribosomes, which account for approximately 50% of the total cellular protein content and for approximately 85% of the total RNA (11). Adjusting ribosome biogenesis and turnover to the available nutrients and energy status is therefore critically important for cell survival. This is reflected by a rapid increase in the number of ribosomes during exponential phase and a decline when cells transition into stationary phase (47) (Table 1). Ribosome biogenesis is primarily determined by the rate of rRNA transcription (42, 48), which is strictly linked to the cellular ATP levels (49, 50). *E. coli* contains seven rRNA operons, each encoding the 16S rRNA, 23S rRNA, 5S rRNA, and variable tRNA genes (51). Each rRNA operon is transcribed from two promotors; the upstream P1 promoter is responsible for high-level rRNA production, while the downstream P2 promoter accounts for basal rRNA production at low growth rates or during stationary phase (51) (Fig. 2). Transcription from the P1 promoter is responsible for a large portion of the global transcriptome in rapidly growing *E. coli* cells (52). Stable binding of RNAP to the P1 promotor of the *E. coli* rRNA operons requires millimolar ATP concentrations and is thus diminished when ATP levels drop during stationary phase (49) (Fig. 2).

**Table 1:** Abundance of ribosomal proteins and ribosome-hibernation factors in *E. coli*

Protein	Protein abundance (proteins/cell)		
	Ribosome profiling data^a^ (45)	Protein mass spectrometry data	
		IBAQ	IBAQ
		Exponential phase^b^ (346)	Stationary phase^c^ (346)
Ribosomal proteins			
uS2 (RpsB)	23,487	13,957	2432
uL4 (RplD)	18,688	11,478	1409
Hibernation factors			
RMF	16,122	138	3538
HPF	2917	2312	4011
RaiA	5010	3045	11,711
Sra	15,792	1835	5851
RsfA/RsfS	796	69	4
YqjD	10,886	2146	6209
ElaB	11,477	2398	4052
YgaM	4734	762	793
EttA	2080	n.d.	n.d.

In general, the protein abundance based on ribosome profiling data is in most cases higher than the data based on protein mass spectrometry. This is potentially explained by the fact that ribosome profiling does not consider protein degradation, which is particularly important for stress-responsive proteins. In addition, these experiments were not performed under identical growth conditions and with identical *E. coli* strains.

Abbreviations: IBAQ, intensity-based absolute quantification; n.d., not determined/not detected.

a *E. coli* MG1655 cells were grown to exponential phase (*A*_600_ = 0.3) in Mops medium with 2% glucose (347).

b *E. coli* BW25113 cells were grown to exponential phase on M9 medium + 5 g/l glucose.

c *E. coli* BW25113 cells were grown to stationary phase (24 h) on M9 medium + 5 g/l glucose.

### Synthesis of (p)ppGpp as a major stress regulator

**Figure 3:**
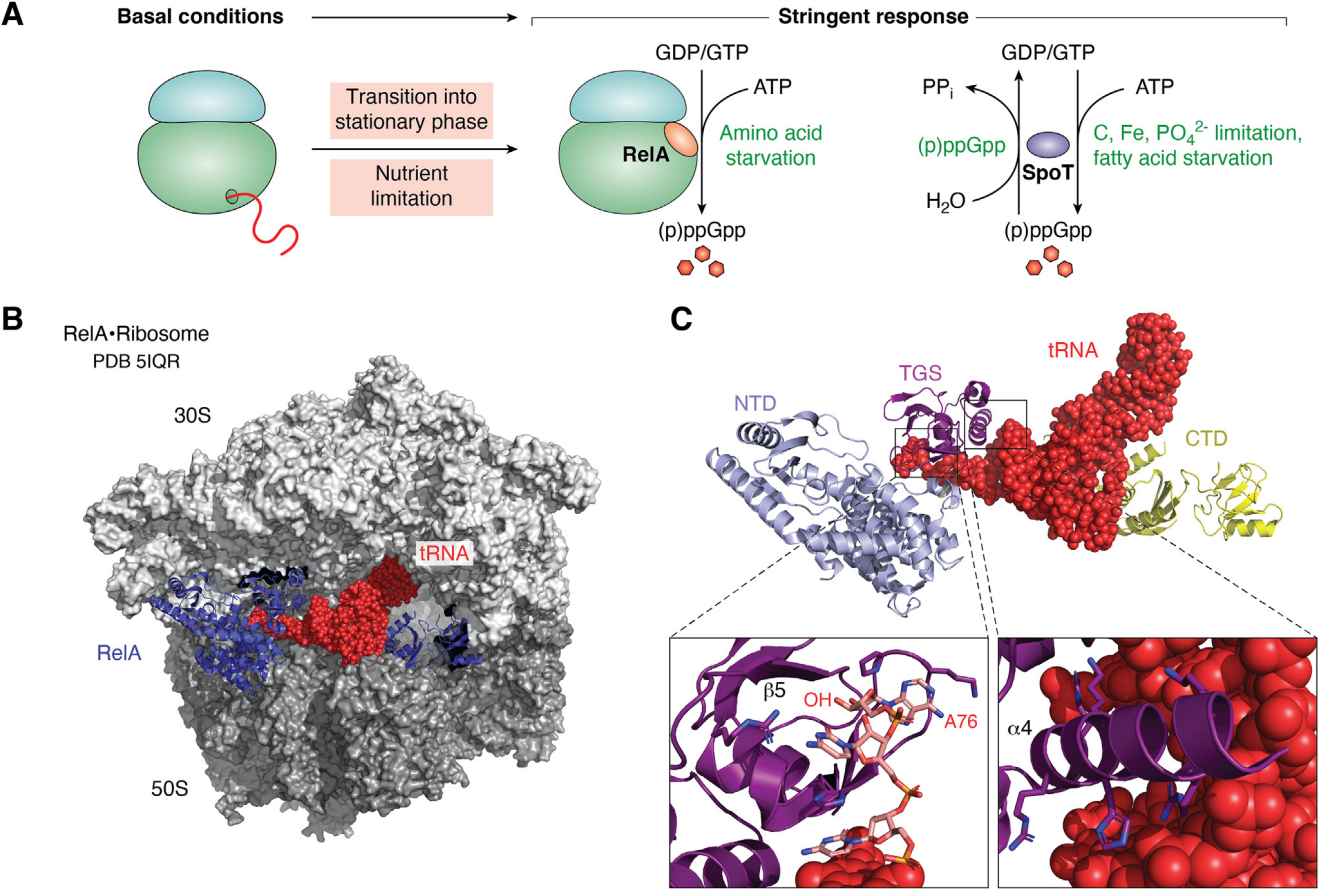
**The stringent response in *E. coli*.***A*, the stringent response in *E. coli* is initiated when cells transition into the stationary phase or encounter nutrient limitation. The (p)ppGpp levels in *E. coli* are mainly controlled by the GDP/GTP pyrophosphokinase RelA and the bifunctional synthetase/hydrolase SpoT. Ribosome-bound RelA synthesizes (p)ppGpp after activation by an uncharged tRNA. (p)ppGpp in turn also induce their own degradation by activation of SpoT. The synthetase activity of SpoT is lower than the synthetase activity of RelA, and (p)ppGpp synthesis by SpoT is induced upon, *e.g.*, fatty acid starvation. *B*, orientation of RelA on the ribosome (PDB 5IQR). The 30S and 50S ribosomal subunits are shown in *light* and *dark gray*, respectively. The uncharged tRNA in the A-site is shown as *spheres in red*, and RelA is shown in *blue*. *C*, zoom-in of the interaction between RelA and the uncharged tRNA. The C-terminal regulatory domain of RelA (CTD; *yellow*) and the TGS domain (*purple*) interact with the uncharged tRNA (*red*) to release the N-terminal catalytic domain (NTD, *light blue*) for (p)ppGpp synthesis (*upper panel*). The 3′ CCA of the tRNA wraps around the TGS domain and interacts with key amino acid residues of RelA. Binding of aminoacetylated tRNAs is prevented because the 3′-OH group (OH) of adenine 76 (A76) of the tRNA is shielded by β-strand 5 (β5) of the TGS domain (*left lower panel*). Positively charged amino acid residues in α-helix 4 (α4) of the TGS domain bind to the phosphate backbone within the acceptor end of the tRNA to further stabilize the RelA–tRNA interaction (*right lower panel*).

Transition into stationary phase is further accompanied by an increase of the polyphosphorylated guanine nucleotides pppGpp and ppGpp by a mechanism termed stringent response (9, 41, 53, 54, 55, 56, 57, 58). The concentrations of (p)ppGpp in *E. coli* are mainly controlled by the GDP/GTP pyrophosphokinase RelA and by the bifunctional synthetase/hydrolase SpoT (41, 59) (Fig. 3*A*). RelA and SpoT show a very similar amino acid sequence and likely evolved *via* gene duplication from an ancestral Rel protein (60), but the hydrolase domain in RelA is inactive (53). Under nonstress conditions, RelA acquires a synthetase-off conformation, in which the N-terminal catalytic domain is inhibited by the C-terminal regulatory domain. However, during amino acid starvation, the regulatory domain interacts with the uncharged tRNA in the A-site of the ribosome, which releases the catalytic domain for (p)ppGpp synthesis (41, 61) (Fig. 3, *B* and *C*). Among the many targets of (p)ppGpp is RelA itself because (p)ppGpp reduce the affinity of RelA for the ribosome, which induces its dissociation from the ribosome. This in turn allows the reactivation of RelA by binding to the next stalled ribosome (53). This “hopping model” postulates that the presence of an uncharged tRNA in the ribosomal A-site provides the signal for RelA activation (53). However, RelA can also interact with uncharged tRNAs in the absence of ribosomes and it is therefore possible that RelA binds to the uncharged tRNA first and then transfers it to the vacant ribosomal A-site (62, 63). Nevertheless, the contact of RelA to the ribosome appears to be required for (p)ppGpp synthesis (64).

(p)ppGpp are synthesized by transferring pyrophosphate from ATP to the 3′-hydroxyl group of GDP or GTP, respectively. Thus, increasing (p)ppGpp concentrations ultimately also lead to a reduction of the cellular ATP levels. The reduction of the cellular ATP levels is further enhanced because (p)ppGpp have been shown to inhibit purine nucleotide synthesis in both gram-positive and gram-negative bacteria (53, 65, 66) and to reduce the F_1_F_0_ ATP synthase expression in *Sinorhizobium meliloti* (67). As a consequence of the reduced ATP levels, the rRNA production is further diminished. An *E. coli* Δ*relA* strain maintains high ATP levels even during stationary phase, which highlights the importance of the stringent response to adjust cellular energy homeostasis during stationary phase (68). (p)ppGpp also regulate their own degradation because the N-terminal hydrolase domain of SpoT becomes available only in the presence of (p)ppGpp (Fig. 3*A*) but is confined in an inactive state when (p)ppGpp are absent (69). Different from RelA, SpoT has only a weak synthetase activity, which is furthermore not induced by amino acid starvation but rather by fatty acid starvation or carbon, iron, and phosphate limitation (11, 41) (Fig. 3*A*).

### The influence of (p)ppGpp on the biogenesis and activity of ribosomes

The ppGpp concentration increases from approximately 40 μM in exponentially growing cells to almost 1 mM when the stringent response is induced (70, 71). Increasing (p)ppGpp levels further reduce rRNA transcription and ribosome biogenesis by an additional mechanism, because a stable binding of RNAP to the rRNA P1 promoter is strongly repressed by the ppGpp-bound transcription factor DksA (72) (Fig. 2). The reduction of the usually very high rRNA transcription by DksA and ppGpp in turn increases the availability of the core RNAP for binding the general stress responsive σ-factor RpoS (σ^38^) (73). RpoS-bound RNAP then controls the transcription of as many as 400 to 500 genes in *E. coli* (43, 74) and thus initiates a global response to nonfavorable conditions.

In addition to reducing rRNA transcription, (p)ppGpp also act as competitive inhibitors of GTPases involved in ribosome maturation, such as Era, RsgA, EngA, RbgA, and ObgE, which impairs ribosome biogenesis further (41) (Fig. 2). Additional ribosome-associated GTPases, such as BipA or YchF, are particularly involved in translation under stress conditions (Fig. 2). BipA (TypA) is a paralog of elongation factor G (EF-G) and widely distributed in bacteria and plants (9, 75), while YchF is a universally conserved member of the Obg subfamily of GTPases (76, 77). Both BipA and YchF have been shown to bind to 70S ribosomes and to the 30S ribosomal subunit, and it has been speculated that they exist in two distinct ribosome complexes, depending on the environmental conditions (78, 79). In addition, both BipA and YchF have been implicated in the selective translation of mRNAs encoding for stress response proteins (78, 80). Although YchF belongs to the family of P-loop GTPases, it preferentially hydrolyses ATP rather than GTP (81), which makes it a potential target of the hyperphosphorylated adenine nucleotides (p)ppApp (82). Whether (p)ppGpp influence ATP or GTP hydrolysis by YchF in bacteria is not known, but it was recently shown that ppGpp inhibits ATP binding to the *Arabidopsis thaliana* homologue AtYchF (83). It therefore appears likely that (p)ppGpp also regulate the activity of the stress-responsive ATPase YchF in *E. coli*. Recent data demonstrate that the translation of mRNAs lacking the canonical ribosome binding site is increased in the absence of YchF (78). In many bacterial species such as *Deinococcus* (84) or *Mycobacterium* (85, 86), these leaderless mRNAs often encode for stress-responsive proteins and the inhibition of YchF by (p)ppGpp might boost their selective translation under stress conditions.

Downregulation of ribosome biogenesis during stress conditions or nutrient depletion is likely the most efficient way of reducing protein synthesis. In addition, ribosomes are degraded during stationary phase and the released amino acids and nucleotides provide nutrients for starved cells (87, 88, 89, 90). Still, for rapid inhibition of protein synthesis, bacteria need to execute additional strategies. One strategy is the direct inhibition of initiation and elongation factors during stress conditions, and the second is the stress-induced modification of the translational machinery.

### Regulation of translation initiation under stress conditions

Translation initiation is the rate-limiting step of protein synthesis and requires the formation of a 30S initiation complex that consists of the 30S ribosomal subunit, the fMet-tRNA^fMet^ (N-formylmethionine-specific tRNA charged with N-formylmethionine), and the initiation factors IF1, IF2, and IF3 (91, 92). In particular, the nucleotide-bound state of IF2 is a critical determinant of translation and either favors or prevents translation (93, 94). IF2 binds (p)ppGpp and GTP with similar affinities, but IF2-ppGpp has a significantly lower ribosome affinity, which leads to reduced translation initiation during stringent response (53, 95) (Fig. 2). However, the affinity of IF2 for GTP and therefore the possible competitive inhibition by (p)ppGpp is also influenced by the particular mRNA to be translated (18). It was shown that the presence of two consecutive hairpin loops close to the translation initiation region within mRNAs, termed *structured enhancer of translation initiation* (SETI), can confer (p)ppGpp tolerance and allow for IF2-dependent translation initiation even in the presence of (p)ppGpp (18). The presence of SETI in mRNAs encoding for stress-induced proteins might explain their selective synthesis upon stress-induced (p)ppGpp synthesis (96). (p)ppGpp can also bind to the elongation factors EF-G and EF-Tu, but their affinities for (p)ppGpp are significantly lower compared with that of IF2 (41), and inhibition of IF2 is most likely the main mechanism of translation inhibition by (p)ppGpp.

The cellular ATP levels also directly influence the elongation cycle of the ribosome during protein synthesis. The *E. coli* translation factor EttA (*energy-dependent translational throttle A*) (Table 1) and its paralogs YheS, YbiT, and Uup are members of the large family of the ABC-F proteins (ATP-binding cassette protein F) (97). They contain two ATP-binding sites (98), and many bacterial ABC-F proteins are involved in ribosome protection against antibiotics (97). EttA interacts with the P-site of the ribosome and inhibits translation elongation (99). The EttA–ribosome interaction is stabilized at high ADP:ATP ratios found in energy-depleted cells, but EttA dissociates from the ribosome at high ATP levels (99). This enables *E. coli* cells to adjust protein synthesis according to the cellular energy status. A very recent cryo-EM study has revealed that the ABC-F proteins regulate the elongation cycle in *E. coli* ribosomes by controlling the geometry of the P-site (100).

### Stress-induced modification of ribosomes and tRNAs

Stress-induced modification of ribosomes results in a certain ribosome heterogeneity, *i.e.*, the presence of different ribosomal subpopulations within a single cell (101, 102). One prominent example for stress-induced modifications is the ribosomal stalk complex, which consists of the ribosomal protein uL10 and two bL7/L12 dimers. The ribosomal stalk is largely responsible for translation factor binding and is highly dynamic (103). Originally described as two distinct proteins, it was later demonstrated that bL7 is an N-terminally acetylated variant of bL12 (Table 2). Acetylation of bL12 and hence the bL7 concentration increases during stationary phase, which stabilizes the ribosomal stalk (104). bL7/L12 dimers are also monomethylated at residue K81, and methylation increases during cold stress (105), which could further contribute to temperature-dependent stalk stabilization. Increasing the overall stability of the ribosome during stress conditions prevents ribosomal damage but potentially also reduces the peptidyltransferase activity (106). Many modifications of ribosomal proteins have been described (Table 2), such as methylation (106), acetylation (105, 107), methylthiolation, or phosphorylation (108, 109, 110). However, in most cases it is unknown whether these modifications are dependent on growth conditions and if they influence the ribosomal activity.

**Table 2:** Modifications of ribosomal proteins in *E. coli*

Modification^a^	30S ribosomal protein(s)	50S ribosomal protein(s)	Detection method	Reference
Methylation	uS11	uL3; uL11; bL7/L12; uL16; bL33	^14^C-methyl labeling	(105, 106)
Acetylation	uS5; bS18	bL7	α-Acetyl lysine antibody	(106, 107)
Phosphorylation	uS3; uS4; uS7; uS11; uS12; uS13; bS18; bS21	uL2; uL3; uL5; uL6; bL7/L12; uL13; uL14; uL16; uL18; bL19; bL21; uL22; bL28; bL31	Phosphoproteomics	(108, 110)
Methylthiolation	uS12		Mass spectrometry	(109, 348)

a Although modifications of ribosomal proteins are well documented, it is in most cases still largely unknown whether these modifications are part of the bacterial stress response and whether they influence the ribosomal activity.

Another source of ribosome heterogeneity is the presence of ribosomal protein paralogs (111). Examples are the nonessential ribosomal proteins bL31 and bL36, which both have one paralog, termed bL31B and bL36B. Both paralogs are upregulated when cells enter stationary phase during which they partially replace the original variants (112). One difference between bL31/bL36 and its paralogs is the absence of cysteine motifs in the latter, which potentially increases their resistance toward oxidative damage.

Stress-induced modifications of tRNAs and translation factors are another strategy to adjust protein synthesis. This has mainly been studied upon oxidative stress, because reactive oxygen species are produced by phagocytes as a defense against bacterial infections (113). Translational fidelity is reduced upon oxidative stress due to impaired editing functions of aminoacyl-tRNA synthetases (114). However, protein mistranslation can provide a survival strategy under stress conditions, *e.g.*, when the cognate amino acid is depleted in stationary phase (115). Mistranslation has also been shown to increase the levels of RpoS (116) and thus to induce the general stress response pathway, providing further protection against stressful conditions. Under nonstress conditions, the RpoS levels are low due to its degradation by the ClpXP protease (43). However, the accumulation of aggregated and misfolded proteins during stress conditions or when translational fidelity is reduced, titrates ClpXP away from RpoS and the RpoS levels increase (114). Another oxidation-sensitive factor is EF-G, which is inactivated by the formation of an intramolecular disulfide bond (117). It was proposed that oxidation of EF-G is used as a regulatory mechanism for reversible translation inhibition under oxidative stress conditions (117, 118).

## Stress-induced ribosome hibernation

**Figure 4:**
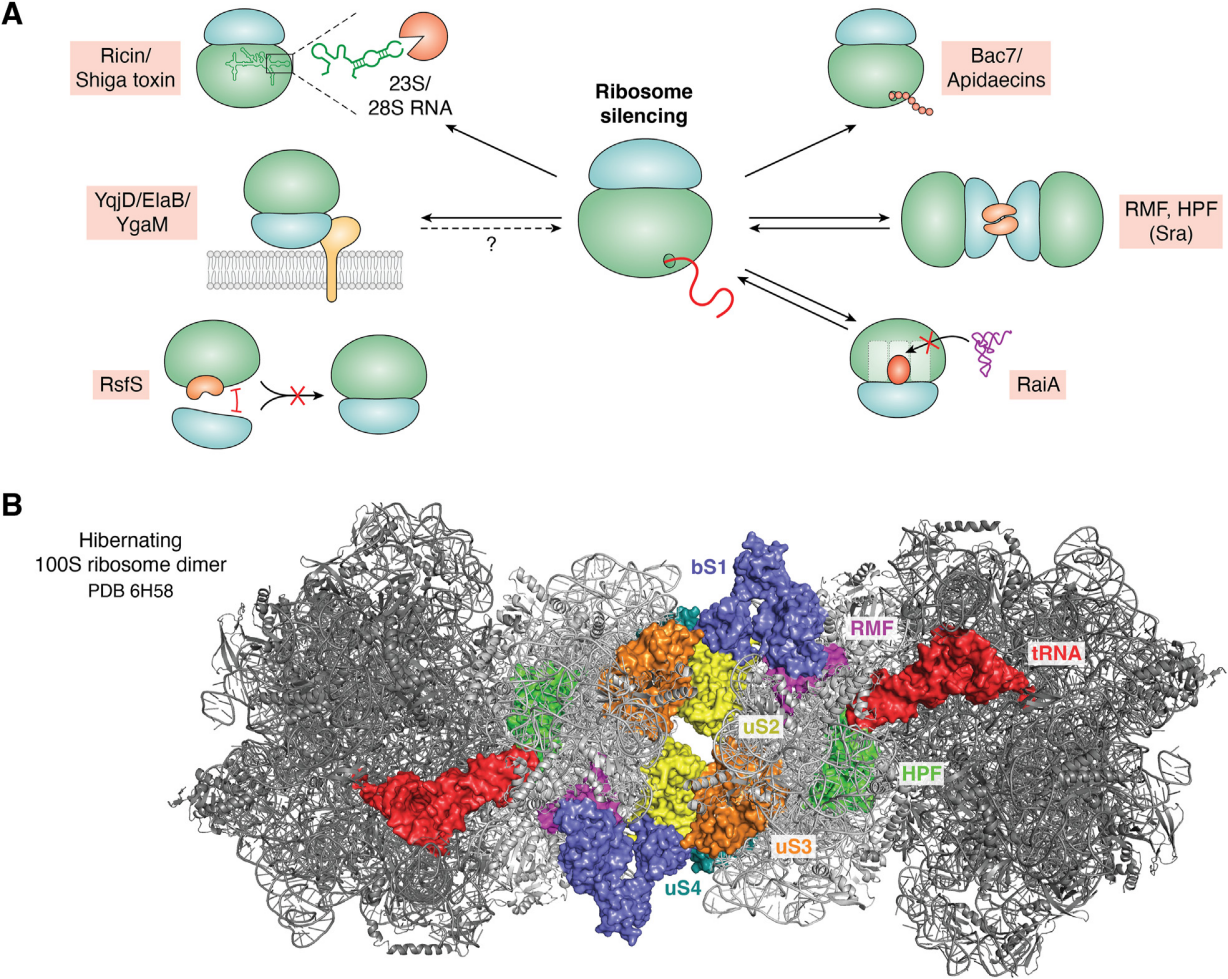
**Ribosome silencing as stress response and defense strategy.***A*, RNA glycosidases, such as Ricin or Shiga toxin irreversibly inactivate ribosomes by cleaving the essential sarcin–ricin loop in 23S or 28S rRNA. Antimicrobial peptides (AMPs) such as Bac7 or apidaecins are secreted by eukaryotic cells and insert into the ribosomal peptide tunnel, leading to ribosome inactivation. Ribosome inactivation by AMPs or Ricin-like enzymes mainly serves as defense mechanism against pathogens and predators. Reversible ribosome inactivation is achieved by multiple proteins, which are expressed in response to stress or starvation conditions. Ribosomes are inactivated by dimerization *via* the coordinated activity of the ribosome modulation factor (RMF) and the hibernation promoting factor (HPF). The activity of RMF is potentially supported by the stationary phase–induced ribosome-associated protein (Sra). RaiA corresponds to a HPF homologue that binds to the P-site of the peptidyl-transferase domain and prevents the access of tRNAs. Yet another mechanism is employed by RsfS, which acts as an anti-association factor and prevents the 50S subunit from joining the 30S subunit. Finally, the three paralogs YqjD, ElaB, and YgaM are so far the only known membrane-bound proteins that have been implicated in ribosome hibernation. However, their mode of action is so far unknown. *B*, cryo-EM structure of the hibernating 100S ribosome dimer (PDB 6H58). The 30S and 50S subunits are colored in *light gray* and *dark gray*, respectively. The binding positions of RMF (*magenta*), HPF (*green*), and the tRNA (*red*) in the E-site of the peptidyltransferase domain are indicated. The localization of the 30S ribosomal proteins, which are involved in the ribosomal contacts, is indicated (bS1 [*blue*], uS2 [*yellow*], uS3 [*bright orange*], and uS4 [*deep teal*]).

Ribosomes are prone to damage when cells enter stationary phase, are exposed to stressful conditions, or are attacked by toxins (9, 119, 120). In particular, an increase in reactive oxygen species can damage the surface-exposed rRNA by inducing chemical modifications of the base or sugar moieties or by generating abasic sites and strand breaks (121). Ribosomal proteins are also sensitive to environmental factors, and their damage is associated with reduced translational fidelity, ribosomal stalling, and protein misfolding (113, 122). Although ribosomes can be repaired, *e.g.*, by replacing damaged ribosomal proteins with functional ones (123) or, as recently shown, by a specific rRNA repair (124), most damaged ribosomes face degradation (125). This, however, would waste all the cellular energy that was initially invested in their biogenesis, and therefore ribosome hibernation is engaged as an additional strategy to protect ribosomes (Fig. 4*A*). Ribosome hibernation was shown to protect ribosomes against RNase-dependent degradation in different bacterial species, such as *Staphylococcus aureus* (126) or *E. coli* (127). Ribosome hibernation also adjusts the pace of protein synthesis to different environmental conditions (9, 11, 12, 13, 14, 20, 127, 128, 129, 130).

The ability to silence ribosomes is present in all domains of life, and both irreversible and reversible mechanisms for ribosome hibernation are employed (14). Irreversible ribosome inactivation can be induced by RNA N-glycosidases, which depurinate adenine residues within the highly conserved sarcin–ricin rRNA loop of both eukaryotic and prokaryotic ribosomes (131). Examples are the plant toxin ricin, produced by *Ricinus communis*; the bacterial Shiga toxin, produced by *Shigella dysenteriae*; or the Shiga-like toxin, produced by pathogenic *E. coli* strains (132, 133, 134) (Fig. 4*A*). These toxins are classified as type II ribosome-inactivating proteins (RIPs), because they consist of two peptide chains: an enzymatically active A-chain that is linked *via* a disulfide bridge to the B-chain. In case of ricin, the B-chain is a galactose-specific lectin that promotes endocytosis of ricin and its subsequent activation *via* disulfide isomerases (131). In general, the irreversible inactivation of ribosomes primarily serves as a defense mechanism against predators or pathogens and is also actively employed by bacterial pathogens (131). Combatting pathogens can also be achieved by proline-rich antimicrobial peptides (AMPs), which are secreted by many eukaryotic cells (120, 135). AMPs, such as Bac7 or apidaecins, target the bacterial ribosome and inhibit translation by inserting into the ribosomal polypeptide tunnel (120, 135) (Fig. 4*A*). In general, ribosome inactivation by type II RIPs and AMPs appears to be less important as a strategy for stress adaptation, although some stress-responsive proteins show similarity to type II RIPs (136).

### Reversible ribosome inactivation during stress conditions

Reversible ribosome inactivation is frequently induced by stress- or growth phase–dependent production of RIPs (12, 14, 19). One of the best-studied examples of reversible RIPs in bacteria is the *ribosome modulation factor* (RMF) (137, 138, 139, 140) (Table 1 and Fig. 4). Together with the *hibernation promoting factor* (HPF), RMF induces the formation of translationally silent 100S ribosome dimers in γ-proteobacteria (14, 129). In the first step, RMF dimerizes the 70S ribosomes to an unstable 90S dimer (137, 141), which is then converted to the more stable 100S dimer by HPF (12, 14, 142, 143). Structural analyses revealed that HPF interacts with the anticodon stem loop of the tRNA in the E-site of the ribosome and that it blocks the mRNA-binding site and access of tRNAs to the A- and P-sites within the peptidyltransferase domain (129, 144). RMF on the other hand, binds to the back of the 30S ribosomal subunit and together with the ribosomal protein bS1 captures the anti-Shine-Dalgarno sequence of the 16S rRNA. In translationally silent 100S ribosomes, HPF and RMF promote contacts between bS1/uS2 of one 30S subunit and uS4/uS3 of the other 30S subunit, thereby aiding stable dimerization as a result of induced conformational changes within the 30S subunits (144) (Fig. 4*B*). Both RMF and HPF are rather small (RMF 55 amino acids, HPF 95 amino acids) and positively charged proteins, which are produced when cells enter stationary phase (127). The expression of *rmf* is positively regulated by (p)ppGpp (137, 145) and by cAMP-CRP, which senses carbon availability (146). The levels of RMF also increase when the SRP receptor FtsY is depleted (35), demonstrating that cells respond to impaired protein targeting by reducing protein synthesis. The expression of *hpf* in *E. coli* is additionally controlled by RpoE (σ^24^), which responds to heat shock and membrane stress and by the autoinducer-2 quorum sensing pheromone (147). *E. coli* also contains a paralog of HPF, termed RaiA (*ribosome-associated inhibitor A*, also called pY or YfiA) (148, 149). Expression of *raiA* is induced (p)ppGpp dependently during stationary phase and under cold stress but repressed by FNR in the absence of oxygen (148, 150). A primary role for FNR is to coordinate carbon and energy metabolism during growth under anaerobic conditions (151) and the repression of *raiA* under anaerobic conditions indicates that RaiA is particularly involved in ribosome hibernation during aerobic conditions. RaiA consists of 113 amino acids and binds to 70S ribosomes where it occupies the P-site of the 30S subunit. This prevents access of the tRNA to both the P- and A-sites and thus blocks translation initiation (152) (Fig. 4*A*). Importantly, while HPF promotes RMF-dependent 100S formation, RaiA prevents it (153). The extended C-terminal tail of RaiA presumably blocks binding of RMF and thereby also RMF-induced ribosome dimer formation (129).

A longer form of HPF (185 amino acids) is also commonly found in gram-positive bacteria and induces 100S ribosome formation in the absence of RMF (142). Structural analyses demonstrate that 100S ribosome dimers in *S. aureus* are formed *via* the dimerization of the C-terminal extensions of the ribosome-bound long HPF (154).

In *E. coli*, 40 to 80% of all ribosomes are assumed to be converted to 100S ribosomes during stationary phase (141, 155). Persister cells, which are highly resistant to antimicrobial drugs and environmental stress, also contain high levels of RMF (156). When conditions improve, hibernating 100S ribosomes are converted into translationally competent 70S ribosomes within minutes (157). However, the molecular mechanisms that lead to 100S dissociation in *E. coli* are still largely unknown, although the involvement of initiation factors IF3 and HflX have been suggested (12, 158).

*E. coli* contains additional stationary phase–induced ribosome-interacting proteins, but their interactions with ribosomes and their physiological functions are still largely unknown (Table 1) (Fig. 4*A*):

**Sra** (*stationary phase–induced ribosome associated*; also termed protein D) is a small (45 amino acids) and basic protein that was identified as a ribosome-associated protein in *E. coli* by two-dimensional PAGE (159). It binds tightly to the 30S subunit and was initially considered to be a ribosomal protein (therefore initially termed S22, *rpsV*) (159). The expression of *sra* is predicted to be controlled by (p)ppGpp and RpoS (159, 160), and the copy number increases when cells enter stationary phase, and also in persister cells (161). The exact function of Sra is unknown, but it was suggested to support the function of RMF (162). A Δ*sra* strain was originally reported to have no phenotype, but a recent study points to increased antibiotic sensitivity when the toxin HipA is expressed in this strain (161). Sra might therefore be important for maintaining persistence.

**RsfS** (*ribosome silencing factor S*, also termed RsfA) in *E. coli* is composed of 105 amino acids and binds to the uL14 protein of the 50S subunit where it likely acts as an anti-association factor that prevents the formation of the translationally active 70S ribosome (163, 164). RsfS has also been suggested to support the GTPase ObgE during ribosome biogenesis and was found to be present in ObgE-containing pre-50S precursors in a recent cryo-EM study (165). The main function of RsfS is likely to prevent the formation of a translationally competent 70S ribosome under nonfavorable conditions, which has also been proposed for the RsfS homologues in *S. aureus* (166) and *Mycobacterium tuberculosis* (164). A Δ*rsfS* strain of *E. coli* is impaired in adapting to stationary phase and is more sensitive to antibiotics (163).

**YqjD** and its paralogs **ElaB** and **YgaM** belong to the small number of C-tail anchored inner membrane proteins in *E. coli* (167) and are predicted to be involved in ribosome hibernation (12, 155). YqjD was shown to associate *via* its N terminus with the 30S subunit in 70S and 100S ribosomes during stationary phase (155). *In vivo*, YqjD is preferentially localized to the cell pole, and it was recently proposed that polar localization is determined by the phosphatidic acid content of the inner membrane (168). Expression of *yqjD*, *elaB*, and *ygaM* is regulated by RpoS (12, 155), and *elaB* expression is additionally controlled by (p)ppGpp and by the transcription factor OxyR, which responds to oxidative stress (169). Proteome analyses indicate that YgaM is significantly less abundant than YqjD or ElaB (Table 1). YqjD and its paralogs are so far the only known *E. coli* membrane proteins that have been associated with ribosome hibernation, but the importance of having both membrane-anchored and soluble RIPs is still unknown.

The importance of protecting ribosomes and adjusting translation during stationary phase or when cells encounter stress conditions is immediately obvious (14), but what is less obvious is why *E. coli* and many other bacteria use so many different proteins to reach this goal (Table 1). Considering the number of ribosomes and the number of (putative) RIPs in *E. coli* cells, it appears that RIPs are much more abundant than ribosomes during stationary phase (Table 1) and that there is a large “overkill.” Why cells invest so much effort in shutting down protein synthesis is not entirely clear. It could be related to the observation that reducing the number of actively translating ribosomes is important for maintaining a constant elongation rate when nutrients are scarce (170). In addition, as recent data indicate that RMF binds to ribosomes only at early stationary phase and is rapidly degraded later during stationary phase (171), it is possible that the different RIPs act at different stages of stationary phase. It is also still unknown whether the different RIPs compete with each other or whether they target different ribosomal subpopulations. Despite the excess of RIPs during stationary phase, many proteins associated with stress tolerance are efficiently produced during stationary phase. This includes not only RIPs as described above but also many additional proteins of the RpoS regulon, such as Dps, which protects DNA and sequesters iron and is one of the most abundant proteins during stationary phase (74, 172). This is also in line with the observation that the translational activity drastically drops when cells enter stationary phase, but is then maintained at a basal level (171).

Hibernation not only is required for protecting ribosomes during stress conditions but is also important for cell differentiation. In zebrafish, it was recently shown that ribosomes transition from a dormant state to an active state within the first hours of embryogenesis (173). The dormant state is induced by four proteins (eIF5a, eEF2, Habp4, and Dap1b), which simultaneously associate with ribosomes in zebrafish eggs and early embryos but are absent on later-stage ribosomes (173). Dap1b is particularly interesting, because it is enriched in basic amino acids and was found to reside inside of the ribosomal peptide tunnel (173). Dap1b could act as a plug that inhibits the peptidyltransferase activity of the ribosome, similar to what has been suggested for the AMP Bac7 (135) or for macrolide antibiotics (174). To which extent ribosome hibernation is also important for cell cycle control in bacteria is currently unknown.

## General concept of protein trafficking in bacteria: the Sec system

Protein trafficking from the bacterial cytosol to the cytoplasmic membrane, the periplasmic space, the outer membrane, or the extracellular environment is an essential part of the proteostasis network, and many secreted proteins are critically important for sensing and balancing stress conditions. One example is the outer membrane lipoprotein RcsF, which senses outer membrane damage and transmits this information to the Rcs signal transduction system (175). Other examples are the periplasmic chaperone/protease DegP, which is associated with thermal, osmotic, and oxidative stress tolerance (176, 177, 178), or the phage-shock proteins, which monitor the integrity of the inner membrane (179).

**Figure 5:**
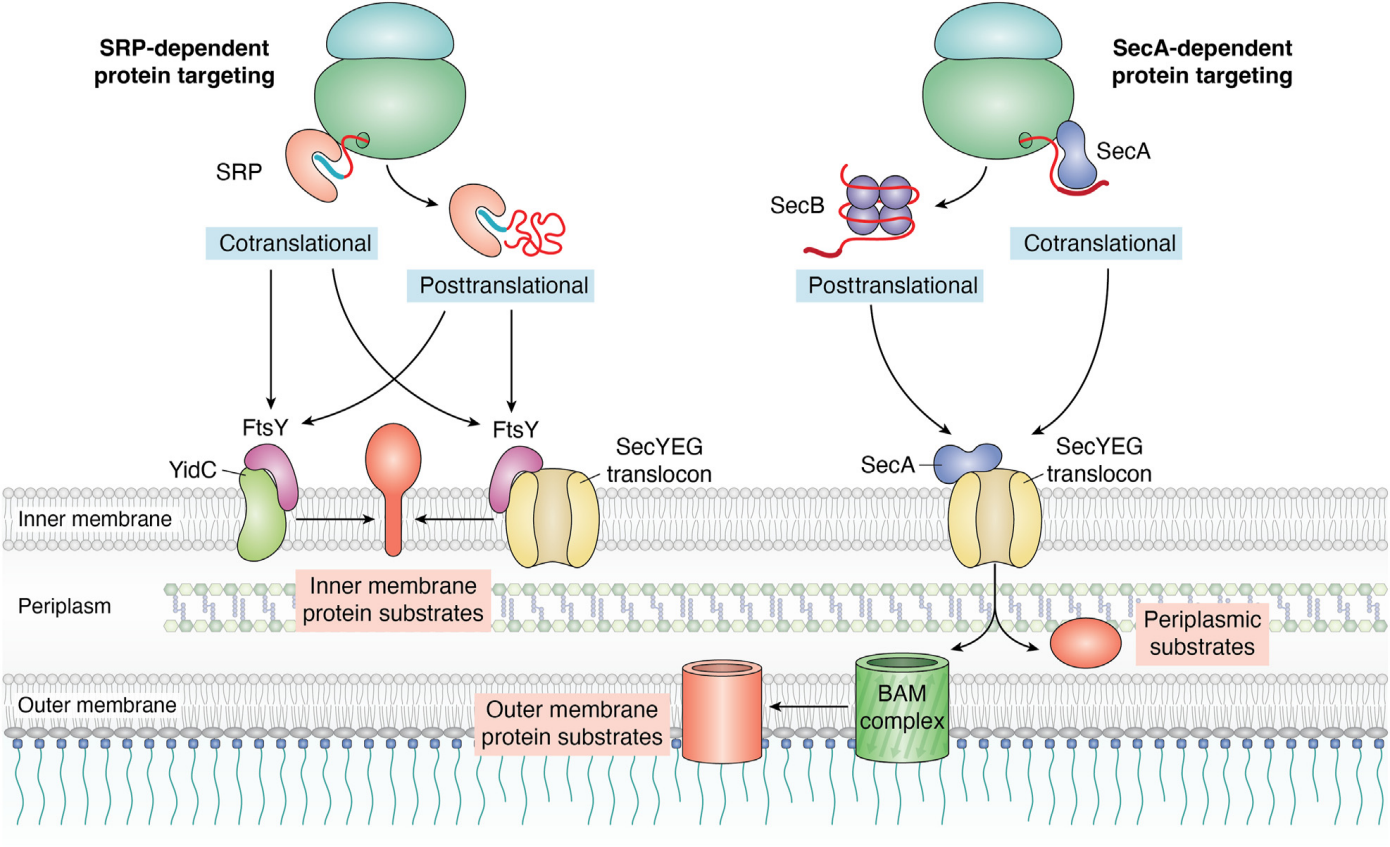
**Protein targeting to the SecYEG translocon and the YidC insertase in *E. coli*.** SRP predominantly recognizes its membrane protein substrates cotranslationally *via* the exposed signal anchor sequence (*blue thick line*) and targets the ribosome-associated nascent chain to the SRP receptor FtsY, which is bound to either SecY or YidC. After docking of the translating ribosome onto SecY or YidC, ongoing translation and lipid partitioning favor membrane insertion of the nascent membrane protein and GTP hydrolysis leads to the dissociation of the SRP–FtsY targeting complex (not shown). SRP can target some membrane proteins also posttranslationally, *i.e.*, after their release from the ribosome. The posttranslational mode is executed for small membrane proteins (<50 amino acids) and is likely also used by C-tail-anchored membrane proteins. SecA primarily targets secretory proteins, *i.e.*, proteins of the periplasm and the outer membrane. These proteins contain cleavable signal sequences (*thick red line*), which are cleaved during protein translocation by signal peptidase (not shown). The majority of SecA is bound to the SecYEG translocon where it serves as substrate receptor and binds substrates posttranslationally. Some substrates are maintained in a transport-competent conformation by chaperones, such as the tetrameric SecB complex. Repetitive ATP-hydrolysis cycles by SecA are required for translocating substrates into the periplasm, where they are further processed by chaperones (not shown) and eventually targeted to the Bam complex for outer membrane insertion. SecA can also interact with the ribosome and recognize some substrates cotranslationally.

The majority of proteins that exit the cytosol to reside in the inner membrane, the periplasm, or the outer membrane are recognized either by the GTPase SRP or the ATPase SecA (38, 180, 181, 182, 183, 184) (Fig. 5). Both SecA and SRP target their substrates to the universally conserved SecYEG translocon in the cytoplasmic membrane (5, 38, 185). The SecYEG translocon in *E. coli* consists of three core subunits: the channel-forming SecY subunit, the SecY-stabilizing SecE subunit, and the dual-topology SecG subunit. In addition, the SecYEG translocon interacts with multiple accessory proteins, which are involved in different steps of transport, such as substrate recognition, substrate release, or folding (5, 38, 186). The general concept of membrane protein insertion *via* the SecYEG translocon predicts that substrates first enter the aqueous SecY channel before they are released into the lipid bilayer *via* the lateral gate of SecY (181, 185, 187, 188). Unexpectedly, however, it was recently shown that, in mammalian cells, some multispanning membrane proteins insert into the membrane without complete prior passing through the homologous Sec61 channel. Instead, after the initial insertion *via* the Sec61 channel, these proteins recruit a membrane-bound protein complex, called MPT (**M**ulti**p**ass **T**ranslocon), which associates with the Sec61 channel (189, 190). Although MPT-like complexes appear to be absent in bacteria, it remains to be analyzed whether indeed all transmembrane domains of a bacterial membrane protein pass through the SecY channel.

SRP can also target some membrane proteins to the YidC insertase, which acts as an additional integration site for membrane proteins in bacteria (191, 192, 193), and also cooperates with the SecYEG translocon during membrane protein insertion (191, 194, 195, 196, 197, 198, 199) (Fig. 5). YidC is a member of the Oxa1 superfamily of proteins (200, 201, 202, 203) and is able to insert small membrane proteins or membrane proteins lacking large periplasmic loops into the bacterial membrane (24, 192). The necessity for a second integration site for membrane proteins in addition to SecYEG translocon is likely explained by the low abundance of the SecYEG translocon (approximately 300 copies per *E. coli* cell (6)) and the rather long occupancy of the SecYEG translocon by the translating ribosome during cotranslational protein transport (180).

### Protein targeting by the SRP pathway

**Figure 6:**
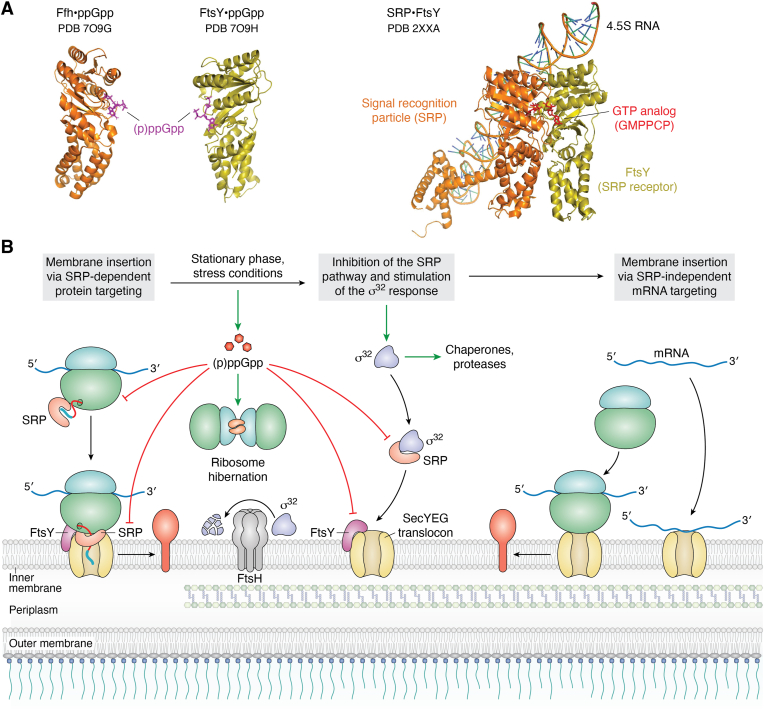
**SRP-dependent and SRP-independent membrane protein insertion in *E. coli.****A*, structures of the ppGpp-bound Ffh (*orange*) and the pppGpp-bound SRP receptor FtsY (*olive*). (p)ppGpp (*magenta*) binding to Ffh and FtsY prevents the formation of the SRP–FtsY targeting complex (*right panel*), which is essential for both co- and posttranslational protein targeting by SRP. The targeting complex consists of the signal recognition particle (Ffh + 4.5S RNA) and its receptor FtsY and is shown in the presence of the GTP analogue GMPPCP (red). Structures were retrieved from the protein database (PDB) with the following IDs: 7O9G (Ffh in complex with ppGpp), 7O9H (FtsY in complex with pppGpp), 2XXA (Signal Recognition Particle [SRP] in complex with its receptor FtsY), and are depicted using Pymol. *B*, schematic representation of SRP-dependent and SRP-independent membrane protein insertion *via* mRNA targeting in *E. coli*. During stationary phase or when cells encounter stress conditions, the (p)ppGpp levels increase, which inhibits the SRP pathway, induces ribosome hibernation, and prevents the SRP-dependent membrane targeting of σ^32^. This reduces σ^32^ degradation by FtsH and stimulates the σ^32^-dependent production of chaperones and proteases. As a consequence of the inhibition of the SRP pathway, some stress-responsive membrane proteins, such as YohP, engage a translation-independent mRNA targeting pathway, which targets mRNAs directly to the SecYEG translocon or YidC, where they are translated by ribosomes and membrane inserted independently of the SRP pathway.

The *E. coli* SRP is composed of the P-loop GTPase subunit Ffh and the 4.5S RNA (180) (Fig. 6*A*). The SRP pathway predominantly targets inner membrane proteins cotranslationally to either the SecYEG translocon or the YidC insertase (6, 192). For cotranslational substrate recognition, SRP binds to the ribosome in close proximity to the ribosomal peptide tunnel exit and scans the ribosomal tunnel for potential substrates (204, 205, 206, 207). After substrate recognition, SRP targets the translating ribosome (ribosome-associated nascent chain, RNC) to the SRP receptor FtsY (208, 209, 210). The GTPase FtsY is exclusively membrane localized in bacteria (211, 212, 213, 214) and has a high affinity for SecY and YidC (191, 215, 216). Binding of the SRP–RNC complex to FtsY aligns the ribosomal peptide tunnel with the SecY channel (215, 217), which then provides a continuous conduit for membrane protein insertion. The subsequent GTP hydrolysis by both Ffh and FtsY leads to the dissociation of the SRP–FtsY targeting complex and allows another targeting cycle (208).

Cotranslational targeting by SRP is limited to substrates of more than 45 to 50 amino acids (218, 219, 220, 221), because only then a sufficient part of the nascent protein is exposed to the outside of the ribosomal tunnel for SRP binding. This excludes the rapidly growing class of small membrane proteins from cotranslational recognition by SRP (120). The production of many of these small membrane proteins is highly upregulated during stress conditions or when cells enter stationary phase (120, 222); YshB, for example, was predicted to be present in more than 11,000 copies in a stationary *E. coli* cell (45). For the 27-amino-acid-long membrane protein YohP and the 33-amino-acid-long membrane protein YkgR, it was recently shown that their insertion still requires the SRP pathway and that SRP targets them posttranslationally to either the SecYEG translocon or the YidC insertase (223) (Fig. 5). This is the first example for a posttranslational function of SRP in bacteria, which is likely also required for the insertion of C-tail anchored membrane proteins in *E. coli*. *E. coli* contains only a small number of proteins that are membrane anchored *via* a C-terminal transmembrane domain (167), but proteins such as the type VI secretion protein TssL (SciP) (224, 225) or the putative RIPs YqjD, ElaB, and YgaM are important for cell survival under stress conditions (225, 226, 227).

### Protein targeting by the SecA pathway

SecA-dependent protein targeting acts primarily on proteins that are delivered to the periplasmic space or to the outer membrane (6, 228). These proteins contain a cleavable N-terminal signal sequence and are mainly recognized by SecA posttranslationally (229) (Fig. 5). In some cases, SecA-dependent substrates are bound by chaperones, such as SecB, after they have been released from the ribosome (230, 231). Like FtsY, SecA is preferentially membrane bound (212, 232), which is due to its affinity for anionic phospholipids and the cytosolic loops of SecY (216, 233, 234, 235). Thus, both SecA and FtsY act as SecYEG-associated substrate receptors for their respective client proteins. However, in contrast to FtsY, SecA does not appear to make contact with YidC (191). Protein translocation across the SecYEG channel depends on repetitive ATP hydrolysis cycles by SecA, and the mechanistic details of this process have been recently described in detail (236, 237, 238). In addition to its posttranslational substrate recognition, SecA can also bind to ribosomes (205, 206, 207, 239) (Fig. 5), which is likely important for the translocation of large periplasmic loops within membrane proteins that are otherwise targeted by the SRP pathway (239, 240, 241). In contrast to the SRP pathway, which is universally conserved, the SecA pathway is only present in bacteria and chloroplasts but lacking in eukaryotes and archaea (5, 242).

## Protein trafficking under stress conditions: the inhibition of protein targeting by alarmones

The nucleotide dependence of SecA, SRP, and FtsY makes them prime candidates for a (p)ppGpp- and (p)ppApp-dependent regulation. Both Ffh and FtsY were identified as potential (p)ppGpp targets in an affinity-based screen (243), which was confirmed in a recent study that demonstrated that ppGpp or pppGpp inhibits both co- and posttranslational targeting by SRP in a concentration-dependent manner (46). Further biochemical and structural studies revealed that (p)ppGpp and GTP bind with similar affinities to the GTPase domains of both Ffh and FtsY (Fig. 6*A*). However, in the ppGpp-bound form of Ffh/FtsY, the δ- and ε-phosphate groups at the 3′-OH group of the ribose prevent the formation of a functional SRP–FtsY targeting complex (46) (Fig. 6*A*). The formation of the SRP–FtsY targeting complex is essential for substrate delivery to the SecYEG translocon or the YidC insertase (215, 244, 245, 246). This suggests that, during stress conditions, when the (p)ppGpp levels increase, protein targeting *via* the SRP pathway is diminished (Fig. 6*B*). The inhibition of the SRP pathway when (p)ppGpp increase during stationary phase or when cells encounter stress conditions is reasonable to adjust the protein transport machinery to the overall reduced protein synthesis rate. However, it potentially also prevents membrane insertion of stress response proteins, which are required under those conditions. This includes the stationary phase–induced small membrane proteins YohP or YkgR; the membrane-bound RIPs ElaB, YqjD, or YgaM; the multidrug-resistant protein MdtF (247); or the cell division protein FtsQ (248, 249). The latter is frequently used as a model substrate for SRP-dependent insertion (221).

The SRP pathway could still be sufficiently active for the insertion of these stress-responsive proteins during stationary phase, because (p)ppGpp do not act as a simple on–off switch of metabolic processes (53). Instead, cells rather execute a priority program of a (p)ppGpp-mediated shutdown, which is determined by the different (p)ppGpp affinities of target enzymes and by the (p)ppGpp/GTP ratios (41, 243). Many proteins involved in amino acid metabolism or translation are inhibited already at submicromolar concentrations (*e.g.*, EF-Tu, IF2, LdcI), while others, such as the DNA primase DnaG, require low millimolar (p)ppGpp levels for inhibition. The K_D_ values of Ffh and FtsY for ppGpp (8–30 μM) and pppGpp (14–70 μM) are in the same K_D_ range as observed for the ribosome assembly factor BipA or enzymes involved in nucleotide metabolism and transcription (41, 46). Thus, inhibition of the SRP pathway apparently occurs already when cells transition from basal (p)ppGpp levels (53) to (p)ppGpp levels that lead to an adaptational program but do not yet shut down metabolism completely (57). Still, the local concentrations of (p)ppGpp in the immediate vicinity of the RelA–ribosome complex are probably high enough to inhibit the ribosome-bound SRP. In addition, due to its low abundance (<300 copies in an *E coli* cell; (6)), SRP is likely an early target of (p)ppGpp-mediated inhibition *in vivo*. This then indeed raises the question of how stress-responsive membrane proteins are membrane inserted when the SRP pathway is inhibited by (p)ppGpp.

The consequences of SRP or FtsY depletion have been analyzed in multiple studies, which collectively show a reduction of the membrane proteome, ultimately leading to cell death (36, 250, 251, 252). Still, the data also indicate that depletion of the SRP pathway does not affect the insertion of all membrane proteins equally, pointing to alternative pathways that can be engaged by some SRP substrates. The depletion of SRP in *E. coli* induces the σ^32^ response, which is linked to an increase of chaperones and proteases, such as DnaK, GroEL, ClpB, and FtsH (27, 36) (Fig. 6*B*). DnaK is involved in the insertion of C-tail anchored membrane proteins (226) and promotes the translocation of secretory proteins when SecA is impaired (28), but DnaK is not able to compensate for a lack of SRP/FtsY during insertion of membrane proteins, as recently shown for YohP insertion (223). Alternatively, some stress-responsive membrane proteins might use SecA, which is upregulated when protein transport *via* the SecYEG translocon is impaired (253, 254). SecA can insert the type II single spanning membrane protein RodZ (255, 256) or single-spanning variants of YidC (240) into the *E. coli* membrane but is unable to insert highly hydrophobic proteins, which include most of the stress-responsive membrane proteins (239). This is also supported by *in vitro* studies using purified inner membrane vesicles or reconstituted SecYEG proteoliposomes, which demonstrate that SecA is unable to promote the insertion of most single or multispanning membrane proteins (211, 223, 252, 257).

Bacteria can potentially cope with a (p)ppGpp-dependent shutdown of the SRP pathway by engaging a translation-independent mRNA targeting pathway (258, 259, 260) (Fig. 6*B*). This is supported by the recent observation that the insertion of the small membrane protein YohP occurs independent of the SRP pathway when YohP is produced from already membrane-bound mRNAs (261). Although details on potential targeting factors that route mRNAs to the membrane are still largely missing, recent data indicate that the SecYEG translocon and the YidC insertase can bind mRNAs (261). This is in agreement with data showing that the homologous Sec61 complex in eukaryotes binds mRNA (262, 263) and also supported by the intrinsic ability of the SecYEG translocon to bind ribosomes primarily *via* the rRNA (264, 265, 266). Thus, the available data indicate that when SecYEG- or YidC-bound mRNAs are translated, the translation product can be inserted into the membrane without the need for SRP and its receptor FtsY (261). Importantly, membrane insertion of YohP is drastically impaired *in vivo* when the stringent response is induced and membrane targeting of the *yohP* mRNA is simultaneously reduced by changing the nucleotide composition of the mRNA (261). This suggests that SRP-dependent targeting and mRNA targeting act in parallel and that mRNA targeting can compensate for impaired SRP-dependent targeting. However, it is currently unknown whether mRNA targeting can also sustain SRP-independent insertion of larger membrane proteins.

The inhibition of the SRP pathway by (p)ppGpp is intriguing in light of the σ^32^ response that is induced in Ffh- or FtsY-depleted *E. coli* cells (38). σ^32^ can compete with the house-keeping σ^70^ for RNAP binding only at elevated (p)ppGpp levels (40), which implies that the (p)ppGpp levels are increased in Ffh- or FtsY-depleted *E. coli* cells. Although this needs to be experimentally verified, it would explain why FtsY-depleted cells show increased levels of RMF (35), which is transcriptionally controlled by the ppGpp levels (267). An increase of (p)ppGpp upon impaired protein transport would reduce the global protein synthesis rate as described above and prevent the cytosolic accumulation of SRP substrates. A reduced translation rate can indeed compensate for a loss of SRP-dependent targeting and delay cell death (268, 269). The depletion of SRP also leads to an upregulation of polyphosphate kinase (36), which synthesizes the chemical chaperone polyphosphate as additional means for stress protection (270).

In addition to (p)ppGpp, bacteria can also produce (p)ppApp, although generally less is known about their synthesis. In *Pseudomonas aeruginosa*, (p)ppApp were recently shown to be produced by the type VI secretion toxin Tas1. Tas1 is secreted into a bacterial target cell where it produces large amounts of (p)ppApp at the expense of ATP (271). The depletion of the cellular ATP pool diminishes cell growth of the target cell and gives *P. aeruginosa* a competitive advantage. In *E. coli*, (p)ppApp were shown to bind to RNAP *in vitro* and to activate transcription of the rRNA promoter (272), which potentially implies that (p)ppGpp and (p)ppApp have opposing effects on the transcription of at least some promoters. However, it is still under debate to which extent (p)ppApp are produced in *E. coli* cells; a two-dimensional thin-layer chromatography approach has identified (p)ppApp in wildtype *E. coli* cells but not in Δ*relA*Δ*spoT* cells (273). A ppApp-based affinity pull-down in *E. coli* identified only six potential targets in the soluble fraction (274); among them is the serine protein kinase YeaG, which is implicated in adaptation to nitrogen starvation (275). So far there are no studies exploring the effect of (p)ppApp on the activity of SecA in the targeting and translocation of secretory proteins. However, it appears unlikely that a potential (p)ppApp-induced inhibition of the SecA pathway can be compensated for by mRNA targeting, as observed for the inhibition of the SRP pathway. This is because SecA is not only required for targeting secretory proteins to the SecYEG translocon but also provides the energy for translocation *via* ATP hydrolysis (237). This is different for the SRP pathway, where GTP hydrolysis is required for the dissociation of the SRP-FtsY targeting complex at the end of the targeting reaction, while the energy for the actual membrane insertion comes from the translational activity of the ribosome during cotranslational insertion and lipid partitioning during co- and posttranslational insertion (276, 277).

## Modulation of protein translocases and insertases by stress conditions

The SecYEG translocon and the YidC insertase are responsible for transporting the majority of proteins into or across the inner membrane (183, 184). Additional membrane-bound transport systems include the twin-arginine transport (Tat) system, which in *E. coli* consists of TatA, TatB, and TatC (6, 278). The Tat system is responsible for transporting folded and cofactor-containing proteins, such as multicopper oxidases, which provide the first line of defense against periplasmic copper (Cu) toxicity (279, 280). Once proteins enter the periplasm, they are further processed by periplasmic chaperones, such as SurA, Skp, or DegP, and proteins designated for the outer membrane are eventually inserted by the Bam complex (281, 282, 283) (Figs. 1 and 5). The SecYEG translocon, the YidC insertase, or the TatABC complex lack nucleotide-binding sites and therefore (p)ppGpp or (p)ppApp cannot directly influence their activity. Likewise, due to the absence of nucleotides in the bacterial periplasm, the periplasmic chaperones and the Bam complex can also not be directly targeted by alarmones.

**Figure 7:**
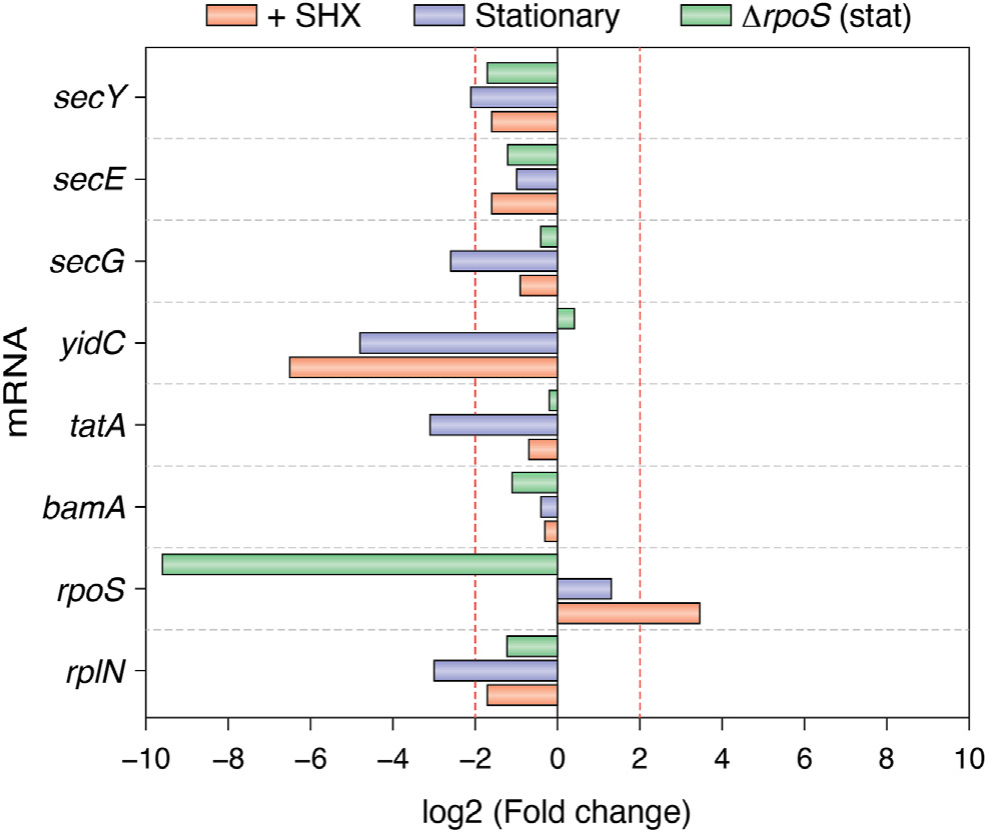
**Transcriptional regulation of genes encoding for protein translocase or insertases in *E. coli.*** Data were retrieved from Gene Expression Omnibus databank (https://www.ncbi.nlm.nih.gov/) with the accession numbers GSE7885 (wildtype *versus* Δ*rpoS)*, GSE 19742 (±SHX), and GSE15534 (logarithmic *versus* stationary phase). Transcriptome data were analyzed by the GEO2R software platform (https://www.ncbi.nlm.nih.gov/geo/geo2r/). SHX refers to serine hydroxamate, an amino acid derivative that is used for inducing stringent response (344). *RplN* encodes for the ribosomal protein uL14 and is encoded in the *spc* operon, where also *secY* is encoded.

Still, because the specificity and activity of RNAP is determined by the (p)ppGpp levels, alarmones could potentially regulate the production of protein translocases and insertases at the transcriptional level. SecY is encoded in the *spc* operon, which encodes for ribosomal proteins and which is negatively regulated by (p)ppGpp and the transcriptional regulator DksA (284). In addition, the *spc* operon is regulated by a translational feedback loop that involves the ribosomal protein uS8, which is part of the *spc* operon (285). However, uS8 does not seem to regulate the production of SecY or bL36, which are encoded by the last two genes of the *spc* operon. There is also no reported effect of (p)ppGpp on *secY* expression, but the deletion of *rpmJ*, encoding bL36, decreases *secY* expression and impairs protein transport (286). *RpmJ* expression is reduced during stationary phase and bL36 is at least partially replaced by its paralog bL36B, which is encoded outside of the *spc* operon (112). Thus, the downregulation of *rpmJ* during stationary phase might reduce *secY* expression. However, transcriptome data of *E. coli* cells grown to stationary phase in the absence or presence of the stress-responsive σ-factor RpoS, or of cells treated with serine hydroxamate, for inducing the stringent response (55), do not show strong changes in the abundance of the *secY* mRNA (Fig. 7). It is likely that an additional promoter within the *spc* operon allows the production of *secY* independently of the other *spc* genes. There is also no strong effect on *secE* expression, which is encoded in one operon together with the transcription termination factor NusG (287). *SecG* is encoded together with *leuU* (*tRNA*^*Leu*^) in one transcription unit (288) and tRNA levels can strongly decline upon stress conditions (289, 290), which potentially explains the slight decrease of the *secG* mRNA when cells are grown to stationary phase (Fig. 7). In general, it appears that the expression of *secY*, *secE*, and *secG* mainly follows the expression of house-keeping genes, without large variations in their steady-state amounts during different growth phases or when exposed to stress conditions (291, 292).

Interestingly, the *yidC* mRNA levels significantly decrease when the stringent response is induced or when cells enter stationary phase in wildtype cells (Fig. 7). This is not observed in the Δ*rpoS* strain, which suggests that *yidC* expression is negatively regulated by RpoS. YidC in *E. coli* is encoded in a conserved gene cluster that contains *rpmH*, *rnpA*, *yidD*, *yidC*, and *trmE* (293). *RpmH* encodes for the ribosomal protein L34, and three upstream promoters control its expression, one of which is sensitive to ppGpp (294, 295). Thus, in contrast to the SecYEG translocon, the YidC insertase is downregulated upon starvation. The requirement for YidC during stationary phase is likely reduced, because many YidC-dependent membrane proteins are involved in energy metabolism, such as the F_1_F_0_-ATPase (296, 297) or the cytochrome *o* oxidase (298), and their abundance is strictly regulated by substrate availability (299, 300).

The *tatABC* operon is under the control of the σ^70^ promoter, which is the main promoter for exponentially growing cells. Genes with promoters that are only recognized by σ^70^ and not by other σ-factors are downregulated during stationary phase or nutrient limitation (301), which explains the decrease of *tatA* mRNA during stationary phase (Fig. 7). Finally, *bamA* transcription is controlled by the heat-responsive RpoE/σ^24^ (302) but does not respond to the stringent response or to stationary phase.

There is also not much known on how the (p)ppGpp levels respond to impaired protein transport, with the exception of the σ^32^ response that is induced upon Ffh or SecE depletion (23, 36) and that depends on (p)ppGpp accumulation (40). However, the depletion of either SecY or BamA does not seem to increase the (p)ppGpp levels, as analyzed by using an *rpoS-mCherry* reporter construct (68). (p)ppGpp act as a positive transcriptional regulator of *rpoS*, and RpoS levels can serve as an indirect indicator of (p)ppGpp levels (43). In contrast, the depletion of LpxA or LptA, which are required for lipopolysaccharide transport to the outer membrane (282, 303), caused a strong increase of the *rpoS-mCherry* fluorescent signal (68). However, considering that LptA is a periplasmic protein that requires the SecYEG translocon for crossing the inner membrane, additional studies are required for analyzing how defects in outer membrane biogenesis influence the (p)ppGpp levels.

Starvation or stress conditions can influence membrane-bound protein translocases and insertases not only directly *via* transcriptional and posttranscriptional mechanisms but also indirectly by changing the lipid composition of the membrane. The phospholipid composition strongly influences the activities of the SecYEG translocon (234, 304, 305, 306, 307), the YidC insertase (308, 309, 310), or the Tat system (311, 312, 313). This is explained by the affinity of the protein targeting factors FtsY and SecA for negatively charged phospholipids (234, 314), which are enriched in close proximity to the SecYEG translocon (306, 307, 315). The length and the saturation of phospholipids are additional factors that influence protein transport. Unsaturated fatty acids have been shown to improve SecA-SecYEG-mediated protein translocation (316), and a hydrophobic mismatch between the short transmembrane domains of TatA or YidC and the phospholipid membrane is critical for protein transport (309, 311, 313). Variations of the phospholipid composition are frequently observed upon temperature changes, *e.g.*, the amount of unsaturated phospholipids strongly increases when cells grow at low temperatures (317, 318). Cells entering stationary phase show an increased proportion of saturated fatty acids, whereas unsaturated fatty acids are almost completely converted to cyclopropane derivatives (319). Both processes have been shown to depend on (p)ppGpp (320) and RpoS (321). The stationary phase is also associated with a global remodeling of the lipid acyl chain structure and with an increase in cardiolipin (319, 322). Whether the increase of cardiolipin compensates for the lack of SecYEG stimulation that is caused by the decline of unsaturated phospholipids is currently unknown. In general, causes and consequences of altered phospholipid compositions are difficult to dissect, because phospholipid alterations can in turn activate multiple stress response pathways and increase the (p)ppGpp levels (317, 323).

## The importance of protein transport for sensing stress

Coordinating the protein synthesis and protein transport machineries saves cellular resources and prevents damage of proteins in a potentially hostile extracytosolic compartment. Still, protein transport is not only required for maintaining the function of the inner and outer membranes as diffusion barriers but is also important for sensing changing environmental conditions (324, 325). A variety of periplasmic proteins function in nonenzymatic sensing of small molecules, in chemotaxis, quorum sensing, or signaling systems (326, 327, 328). One particular sensing mechanism is used by secreted proteins that contain a ribosomal stalling sequence. This was first shown for SecM, which monitors the translocation activity of the SecYEG translocon (253). SecM is a secreted protein that contains a C-terminal ribosomal stalling sequence that causes a transient elongation arrest (329). However, the translocation activity of the SecYEG translocon is sufficient to relieve the elongation arrest and SecM is secreted into the periplasm, where it is degraded by C-terminal-tail-specific protease Prc (329). In contrast, when SecYEG-dependent translocation is impaired, ribosomal stalling persists and the helicase activity of the ribosome unfolds a stem–loop sequence that shields the ribosome-binding site of the downstream-encoded *secA* (254). This boosts SecA production, which in turn stimulates SecYEG-dependent translocation. A similar system is used to control the production of the SecYEG-associated SecDF complex in response to sodium concentrations in *Vibrio cholerae* (330, 331). For DUF2946-like proteins, which are widely distributed in bacteria, it was recently shown that they function as secreted sensor proteins that monitor the periplasmic concentrations of Cu and potentially other toxic heavy metals (332, 333). The DUF2946-like protein CutF was shown to be cotranslationally targeted to the SecYEG translocon by SRP. In the presence of Cu, the C-terminal stalling sequence induces an elongation arrest that allows the unfolding of a downstream-located mRNA stem–loop. This makes the ribosome-binding site of the downstream-encoded multicopper oxidase CutO available and CutO levels increase, which in turn leads to the conversion of Cu(I) to the less toxic Cu(II) (280, 332, 334). Considering the abundance of DUF2946-like proteins in bacteria, translational control by an upstream-encoded and exported sensor protein is likely commonly executed in bacteria.

## Conclusion and outlook

The bacterial stress adaptation is a highly orchestrated response that essentially effects every cellular process. Although multiple systems that sense different intra- and extracellular cues have been identified and characterized, novel mechanisms such as the DUF2946-like heavy metal sensors continue to emerge (332) and demonstrate the genuine complexity of stress response mechanisms. There is a significant redundancy between different stress response pathways, and some targets are regulated by different stress response pathways. This is best exemplified by the protein synthesis machinery, which is targeted by the oxidative stress response, the heat shock response, the stringent response, and alternative σ-factors. This redundancy highlights that adjusting the rate of protein synthesis is of crucial importance for stress adaptation. On the other hand, some pathways appear to be exclusively targeted by only one stress response pathway; *e.g.*, the essential SRP-dependent targeting pathway appears to be exclusively inhibited by the stringent response pathway. However, the SRP pathway has also been implicated in targeting σ^32^ to the membrane for degradation by FtsH (335) (Fig. 6*B*). The accumulation of (p)ppGpp therefore boosts the σ^32^ response, because it reduces the degradation of σ^32^ and favors its competition with σ^70^ for binding to the core RNAP (40). The σ^32^ response is potentially further stimulated, because depletion of SRP increases the concentration of polyphosphate kinase, which synthesizes the chemical chaperone polyphosphate (36). Increased polyphosphate levels stimulate the protease Lon, which degrades Ffh, the protein component of SRP (336). Some reports also indicate that (p)ppGpp inhibit exopolyphosphatase, which degrades polyphosphate (270, 337), although (p)ppGpp might not act directly on exopolyphosphatase (338). Nevertheless, this emphasizes the enormous cooperativity between the different stress response pathways, which needs to be further explored.

Nucleotides exist in different forms and act at the center of metabolic regulation, but besides (p)ppGpp, their roles in regulating protein synthesis or protein targeting have barely been touched. This applies to (p)ppApp, and also to the ubiquitous dinucleoside polyphosphates (Np_n_Ns; where N can correspond to adenine, uridine, or cytosine and n represents the number of phosphates) (42). The diadenosine tetraphosphate (Ap_4_A) levels strongly increase when cells are exposed to oxidative stress or to aminoglycoside antibiotics (339), and it was shown that Ap_4_A attached to the 5′-end of mRNAs increases their stabilities (340). Whether this also has a direct influence on their translation by the ribosome or whether dedicated ribosomes translate these capped mRNAs requires further analyses.

Finally, the diversity of sensing and response systems provides a major evolutionary advantage and enables bacteria to survive astonishingly harsh conditions and to potentially escape antimicrobial treatments. The bacterial stress response is therefore an attractive target for antimicrobial compounds (341). One example is relacin, a 2′-deoxyguanosine-based analogue of ppGpp, which inhibits (p)ppGpp synthesis and leads to cell death (342). Some peptides (343, 344) or the compound X9, identified in a high-throughput screen for potent *M. tuberculosis* Rel inhibitors (345), also targets the stringent response and prevents (p)ppGpp accumulation. Further studies on compounds that inhibit individual stress response pathways will ultimately help to reveal the enormous plasticity of these pathways and will potentially identify novel avenues for antimicrobial therapies.

## Conflict of interest

The authors declare that they have no conflicts of interest with the contents of this article.

## References

[1] Mogk A., Huber D., Bukau B. (2011). Integrating protein homeostasis strategies in prokaryotes. Cold Spring Harb. Perspect. Biol..

[2] Powers E.T., Powers D.L., Gierasch L.M. (2012). FoldEco: a model for proteostasis in E. coli. Cell Rep..

[3] Bittner L.M., Arends J., Narberhaus F. (2017). When, how and why? regulated proteolysis by the essential FtsH protease in Escherichia coli. Biol. Chem..

[4] Rosenzweig R., Nillegoda N.B., Mayer M.P., Bukau B. (2019). The Hsp70 chaperone network. Nat. Rev. Mol. Cell Biol..

[5] Denks K., Vogt A., Sachelaru I., Petriman N.A., Kudva R., Koch H.G. (2014). The Sec translocon mediated protein transport in prokaryotes and eukaryotes. Mol. Membr. Biol..

[6] Kudva R., Denks K., Kuhn P., Vogt A., Muller M., Koch H.G. (2013). Protein translocation across the inner membrane of Gram-negative bacteria: the Sec and Tat dependent protein transport pathways. Res. Microbiol..

[7] Gloge F., Becker A.H., Kramer G., Bukau B. (2014). Co-translational mechanisms of protein maturation. Curr. Opin. Struct. Biol..

[8] Rodnina M.V., Wintermeyer W. (2016). Protein elongation, co-translational folding and targeting. J. Mol. Biol..

[9] Starosta A.L., Lassak J., Jung K., Wilson D.N. (2014). The bacterial translation stress response. FEMS Microbiol. Rev..

[10] Albert B., Kos-Braun I.C., Henras A.K., Dez C., Rueda M.P., Zhang X. (2019). A ribosome assembly stress response regulates transcription to maintain proteome homeostasis. Elife.

[11] Zegarra V., Bedrunka P., Bange G., Czech L. (2023). How to save a bacterial ribosome in times of stress. Semin. Cell Dev. Biol..

[12] Maki Y., Yoshida H. (2021). Ribosomal hibernation-associated factors in Escherichia coli. Microorganisms.

[13] Matzov D., Bashan A., Yap M.F., Yonath A. (2019). Stress response as implemented by hibernating ribosomes: a structural overview. FEBS J..

[14] Prossliner T., Skovbo Winther K., Sørensen M.A., Gerdes K. (2018). Ribosome hibernation. Annu. Rev. Genet..

[15] Bakshi S., Choi H., Weisshaar J.C. (2015). The spatial biology of transcription and translation in rapidly growing Escherichia coli. Front. Microbiol..

[16] Beck H.J., Moll I. (2018). Leaderless mRNAs in the spotlight: ancient but not outdated. Microbiol. Spectr..

[17] Cheng-Guang H., Gualerzi C.O. (2020). The ribosome as a switchboard for bacterial stress response. Front. Microbiol..

[18] Vinogradova D.S., Zegarra V., Maksimova E., Nakamoto J.A., Kasatsky P., Paleskava A. (2020). How the initiating ribosome copes with ppGpp to translate mRNAs. PLoS Biol..

[19] Trösch R., Willmund F. (2019). The conserved theme of ribosome hibernation: from bacteria to chloroplasts of plants. Biol. Chem..

[20] Wang T., Liang C., Zheng M., Liu L., An Y., Xu H. (2020). Ribosome hibernation as a stress response of bacteria. Protein Pept. Lett..

[21] Mahmoud S.A., Chien P. (2018). Regulated proteolysis in bacteria. Annu. Rev. Biochem..

[22] Bukau B., Weissman J., Horwich A. (2006). Molecular chaperones and protein quality control. Cell.

[23] Baars L., Wagner S., Wickstrom D., Klepsch M., Ytterberg A.J., van Wijk K.J. (2008). Effects of SecE depletion on the inner and outer membrane proteomes of Escherichia coli. J. Bacteriol..

[24] Wickstrom D., Wagner S., Simonsson P., Pop O., Baars L., Ytterberg A.J. (2011). Characterization of the consequences of YidC depletion on the inner membrane proteome of E. coli using 2D blue native/SDS-PAGE. J. Mol. Biol..

[25] Wang P., Kuhn A., Dalbey R.E. (2010). Global change of gene expression and cell physiology in YidC-depleted Escherichia coli. J. Bacteriol..

[26] Price C.E., Otto A., Fusetti F., Becher D., Hecker M., Driessen A.J. (2010). Differential effect of YidC depletion on the membrane proteome of Escherichia coli under aerobic and anaerobic growth conditions. Proteomics.

[27] Bernstein H.D., Hyndman J.B. (2001). Physiological basis for conservation of the signal recognition particle targeting pathway in Escherichia coli. J. Bacteriol..

[28] Qi H.Y., Hyndman J.B., Bernstein H.D. (2002). DnaK promotes the selective export of outer membrane protein precursors in SecA-deficient Escherichia coli. J. Biol. Chem..

[29] Baars L., Ytterberg A.J., Drew D., Wagner S., Thilo C., van Wijk K.J. (2006). Defining the role of the Escherichia coli chaperone SecB using comparative proteomics. J. Biol. Chem..

[30] Ito K., Akiyama Y. (2005). Cellular functions, mechanism of action, and regulation of FtsH protease. Annu. Rev. Microbiol..

[31] van Stelten J., Silva F., Belin D., Silhavy T.J. (2009). Effects of antibiotics and a proto-oncogene homolog on destruction of protein translocator SecY. Science.

[32] Herskovits A.A., Shimoni E., Minsky A., Bibi E. (2002). Accumulation of endoplasmic membranes and novel membrane-bound ribosome-signal recognition particle receptor complexes in Escherichia coli. J. Cell Biol..

[33] Arechaga I., Miroux B., Karrasch S., Huijbregts R., de Kruijff B., Runswick M.J. (2000). Characterisation of new intracellular membranes in Escherichia coli accompanying large scale over-production of the b subunit of F(1)F(o) ATP synthase. FEBS Lett..

[34] Weiner J.H., Lemire B.D., Elmes M.L., Bradley R.D., Scraba D.G. (1984). Overproduction of fumarate reductase in Escherichia coli induces a novel intracellular lipid-protein organelle. J. Bacteriol..

[35] Bürk J., Weiche B., Wenk M., Boy D., Nestel S., Heimrich B. (2009). Depletion of the signal recognition particle receptor inactivates ribosomes in Escherichia coli. J. Bacteriol..

[36] Wickstrom D., Wagner S., Baars L., Ytterberg A.J., Klepsch M., van Wijk K.J. (2011). Consequences of depletion of the signal recognition particle in Escherichia coli. J. Biol. Chem..

[37] Wagner S., Baars L., Ytterberg A.J., Klussmeier A., Wagner C.S., Nord O. (2007). Consequences of membrane protein overexpression in Escherichia coli. Mol. Cell. Proteomics.

[38] Oswald J., Njenga R., Natriashvili A., Sarmah P., Koch H.G. (2021). The dynamic SecYEG translocon. Front. Mol. Biosci..

[39] Guo M.S., Gross C.A. (2014). Stress-induced remodeling of the bacterial proteome. Curr. Biol..

[40] Jishage M., Kvint K., Shingler V., Nyström T. (2002). Regulation of sigma factor competition by the alarmone ppGpp. Genes Dev..

[41] Steinchen W., Zegarra V., Bange G. (2020). ppGpp: magic modulators of bacterial physiology and metabolism. Front. Microbiol..

[42] Leiva L.E., Zegarra V., Bange G., Ibba M. (2023). At the crossroad of nucleotide dynamics and protein synthesis in bacteria. Microbiol. Mol. Biol. Rev..

[43] Gottesman S. (2019). Trouble is coming: signaling pathways that regulate general stress responses in bacteria. J. Biol. Chem..

[44] Milo R. (2013). What is the total number of protein molecules per cell volume? a call to rethink some published values. Bioessays.

[45] Li G.W., Burkhardt D., Gross C., Weissman J.S. (2014). Quantifying absolute protein synthesis rates reveals principles underlying allocation of cellular resources. Cell.

[46] Czech L., Mais C.N., Kratzat H., Sarmah P., Giammarinaro P., Freibert S.A. (2022). Inhibition of SRP-dependent protein secretion by the bacterial alarmone (p)ppGpp. Nat. Commun..

[47] Failmezger J., Ludwig J., Nieß A., Siemann-Herzberg M. (2017). Quantifying ribosome dynamics in Escherichia coli using fluorescence. FEMS Microbiol. Lett..

[48] Nomura M. (1999). Regulation of ribosome biosynthesis in Escherichia coli and Saccharomyces cerevisiae: diversity and common principles. J. Bacteriol..

[49] Deng Y., Beahm D.R., Ionov S., Sarpeshkar R. (2021). Measuring and modeling energy and power consumption in living microbial cells with a synthetic ATP reporter. BMC Biol..

[50] Schneider D.A., Gaal T., Gourse R.L. (2002). NTP-sensing by rRNA promoters in Escherichia coli is direct. Proc. Natl. Acad. Sci. U. S. A..

[51] Maeda M., Shimada T., Ishihama A. (2015). Strength and regulation of seven rRNA promoters in Escherichia coli. PLoS One.

[52] Dennis P.P., Ehrenberg M., Bremer H. (2004). Control of rRNA synthesis in Escherichia coli: a systems biology approach. Microbiol. Mol. Biol. Rev..

[53] Bange G., Brodersen D.E., Liuzzi A., Steinchen W. (2021). Two P or not two P: understanding regulation by the bacterial second messengers (p)ppGpp. Annu. Rev. Microbiol..

[54] Bennison D.J., Irving S.E., Corrigan R.M. (2019). The impact of the stringent response on TRAFAC GTPases and prokaryotic ribosome assembly. Cells.

[55] Ferullo D.J., Lovett S.T. (2008). The stringent response and cell cycle arrest in Escherichia coli. PLoS Genet..

[56] Irving S.E., Choudhury N.R., Corrigan R.M. (2020). The stringent response and physiological roles of (pp)pGpp in bacteria. Nat. Rev. Microbiol..

[57] Potrykus K., Cashel M. (2008). (p)ppGpp: still magical?. Annu. Rev. Microbiol..

[58] Hauryliuk V., Atkinson G.C., Murakami K.S., Tenson T., Gerdes K. (2015). Recent functional insights into the role of (p)ppGpp in bacterial physiology. Nat. Rev. Microbiol..

[59] Pausch P., Abdelshahid M., Steinchen W., Schäfer H., Gratani F.L., Freibert S.A. (2020). Structural basis for regulation of the opposing (p)ppGpp synthetase and hydrolase within the stringent response orchestrator Rel. Cell Rep..

[60] Mittenhuber G. (2001). Comparative genomics and evolution of genes encoding bacterial (p)ppGpp synthetases/hydrolases (the Rel, RelA and SpoT proteins). J. Mol. Microbiol. Biotechnol..

[61] Brown A., Fernández I.S., Gordiyenko Y., Ramakrishnan V. (2016). Ribosome-dependent activation of stringent control. Nature.

[62] Winther K.S., Roghanian M., Gerdes K. (2018). Activation of the stringent response by loading of RelA-tRNA complexes at the ribosomal A-site. Mol. Cell.

[63] Kushwaha G.S., Bange G., Bhavesh N.S. (2019). Interaction studies on bacterial stringent response protein RelA with uncharged tRNA provide evidence for its prerequisite complex for ribosome binding. Curr. Genet..

[64] Li W., Bouveret E., Zhang Y., Liu K., Wang J.D., Weisshaar J.C. (2016). Effects of amino acid starvation on RelA diffusive behavior in live Escherichia coli. Mol. Microbiol..

[65] Wang B., Grant R.A., Laub M.T. (2020). ppGpp coordinates nucleotide and amino-acid synthesis in E. coli during starvation. Mol. Cell.

[66] Anderson B.W., Schumacher M.A., Yang J., Turdiev A., Turdiev H., Schroeder J.W. (2022). The nucleotide messenger (p)ppGpp is an anti-inducer of the purine synthesis transcription regulator PurR in Bacillus. Nucleic Acids Res..

[67] Krol E., Becker A. (2011). ppGpp in Sinorhizobium meliloti: biosynthesis in response to sudden nutritional downshifts and modulation of the transcriptome. Mol. Microbiol..

[68] Roghanian M., Semsey S., Løbner-Olesen A., Jalalvand F. (2019). (p)ppGpp-mediated stress response induced by defects in outer membrane biogenesis and ATP production promotes survival in Escherichia coli. Sci. Rep..

[69] Tamman H., Ernits K., Roghanian M., Ainelo A., Julius C., Perrier A. (2023). Structure of SpoT reveals evolutionary tuning of catalysis via conformational constraint. Nat. Chem. Biol..

[70] Varik V., Oliveira S.R.A., Hauryliuk V., Tenson T. (2017). HPLC-based quantification of bacterial housekeeping nucleotides and alarmone messengers ppGpp and pppGpp. Sci. Rep..

[71] Zborníková E., Knejzlík Z., Hauryliuk V., Krásný L., Rejman D. (2019). Analysis of nucleotide pools in bacteria using HPLC-MS in HILIC mode. Talanta.

[72] Shin Y., Qayyum M.Z., Pupov D., Esyunina D., Kulbachinskiy A., Murakami K.S. (2021). Structural basis of ribosomal RNA transcription regulation. Nat. Commun..

[73] Girard M.E., Gopalkrishnan S., Grace E.D., Halliday J.A., Gourse R.L., Herman C. (2018). DksA and ppGpp regulate the σ(S) stress response by activating promoters for the small RNA DsrA and the anti-adapter protein IraP. J. Bacteriol..

[74] Schellhorn H.E. (2020). Function, evolution, and composition of the RpoS regulon in Escherichia coli. Front. Microbiol..

[75] Gibbs M.R., Moon K.M., Warner B.R., Chen M., Bundschuh R., Foster L.J. (2020). Functional analysis of BipA in E. coli reveals the natural plasticity of 50S subunit assembly. J. Mol. Biol..

[76] Landwehr V., Milanov M., Hong J., Koch H.G. (2021). The role of the universally conserved ATPase YchF/ola1 in translation regulation during cellular stress. Microorganisms.

[77] Verstraeten N., Fauvert M., Versees W., Michiels J. (2011). The universally conserved prokaryotic GTPases. Microbiol. Mol. Biol. Rev..

[78] Landwehr V., Milanov M., Angebauer L., Hong J., Jüngert G., Hiersemenzel A. (2021). The universally conserved ATPase YchF regulates translation of leaderless mRNA in response to stress conditions. Front. Mol. Biosci..

[79] deLivron M.A., Makanji H.S., Lane M.C., Robinson V.L. (2009). A novel domain in translational GTPase BipA mediates interaction with the 70S ribosome and influences GTP hydrolysis. Biochemistry.

[80] Ero R., Kumar V., Chen Y., Gao Y.G. (2016). Similarity and diversity of translational GTPase factors EF-G, EF4, and BipA: from structure to function. RNA Biol..

[81] Koller-Eichhorn R., Marquardt T., Gail R., Wittinghofer A., Kostrewa D., Kutay U. (2007). Human OLA1 defines an ATPase subfamily in the Obg family of GTP-binding proteins. J. Biol. Chem..

[82] Anderson B.W., Fung D.K., Wang J.D. (2021). Regulatory themes and variations by the stress-signaling nucleotide alarmones (p)ppGpp in bacteria. Annu. Rev. Genet..

[83] Cheung M.Y., Li X., Ku Y.S., Chen Z., Lam H.M. (2022). Co-crystalization reveals the interaction between AtYchF1 and ppGpp. Front. Mol. Biosci..

[84] de Groot A., Roche D., Fernandez B., Ludanyi M., Cruveiller S., Pignol D. (2014). RNA sequencing and proteogenomics reveal the importance of leaderless mRNAs in the radiation-tolerant bacterium Deinococcus deserti. Genome Biol. Evol..

[85] Shell S.S., Wang J., Lapierre P., Mir M., Chase M.R., Pyle M.M. (2015). Leaderless transcripts and small proteins are common features of the mycobacterial translational landscape. PLoS Genet..

[86] Grabowska A.D., Andreu N., Cortes T. (2021). Translation of a leaderless reporter is robust during exponential growth and well sustained during stress conditions in Mycobacterium tuberculosis. Front. Microbiol..

[87] Basturea G.N., Zundel M.A., Deutscher M.P. (2011). Degradation of ribosomal RNA during starvation: comparison to quality control during steady-state growth and a role for RNase PH. RNA.

[88] Deutscher M.P. (2009). Maturation and degradation of ribosomal RNA in bacteria. Prog. Mol. Biol. Transl. Sci..

[89] Zundel M.A., Basturea G.N., Deutscher M.P. (2009). Initiation of ribosome degradation during starvation in Escherichia coli. RNA.

[90] Arends J., Griego M., Thomanek N., Lindemann C., Kutscher B., Meyer H.E. (2018). An integrated proteomic approach uncovers novel substrates and functions of the lon protease in Escherichia coli. Proteomics.

[91] Myasnikov A.G., Simonetti A., Marzi S., Klaholz B.P. (2009). Structure-function insights into prokaryotic and eukaryotic translation initiation. Curr. Opin. Struct. Biol..

[92] Milón P., Maracci C., Filonava L., Gualerzi C.O., Rodnina M.V. (2012). Real-time assembly landscape of bacterial 30S translation initiation complex. Nat. Struct. Mol. Biol..

[93] Milon P., Konevega A.L., Gualerzi C.O., Rodnina M.V. (2008). Kinetic checkpoint at a late step in translation initiation. Mol. Cell.

[94] Milón P., Rodnina M.V. (2012). Kinetic control of translation initiation in bacteria. Crit. Rev. Biochem. Mol. Biol..

[95] Milon P., Tischenko E., Tomsic J., Caserta E., Folkers G., La Teana A. (2006). The nucleotide-binding site of bacterial translation initiation factor 2 (IF2) as a metabolic sensor. Proc. Natl. Acad. Sci. U. S. A..

[96] Amitai S., Kolodkin-Gal I., Hananya-Meltabashi M., Sacher A., Engelberg-Kulka H. (2009). Escherichia coli MazF leads to the simultaneous selective synthesis of both “death proteins” and “survival proteins”. PLoS Genet..

[97] Fostier C.R., Monlezun L., Ousalem F., Singh S., Hunt J.F., Boël G. (2021). ABC-F translation factors: from antibiotic resistance to immune response. FEBS Lett..

[98] Boël G., Smith P.C., Ning W., Englander M.T., Chen B., Hashem Y. (2014). The ABC-F protein EttA gates ribosome entry into the translation elongation cycle. Nat. Struct. Mol. Biol..

[99] Chen B., Boël G., Hashem Y., Ning W., Fei J., Wang C. (2014). EttA regulates translation by binding the ribosomal E site and restricting ribosome-tRNA dynamics. Nat. Struct. Mol. Biol..

[100] Singh S., Gentry R.C., Fu F., Bailey N.A., Altomare C.G., Wong K.-H. (2023). Cryo-EM studies of the four E. coli paralogs establish ABCF proteins as master plumber of the peptidyl-transferase center of the ribosome. bioRxiv.

[101] Byrgazov K., Vesper O., Moll I. (2013). Ribosome heterogeneity: another level of complexity in bacterial translation regulation. Curr. Opin. Microbiol..

[102] Genuth N.R., Barna M. (2018). The discovery of ribosome heterogeneity and its implications for gene regulation and organismal life. Mol. Cell.

[103] Diaconu M., Kothe U., Schlünzen F., Fischer N., Harms J.M., Tonevitsky A.G. (2005). Structural basis for the function of the ribosomal L7/12 stalk in factor binding and GTPase activation. Cell.

[104] Gordiyenko Y., Deroo S., Zhou M., Videler H., Robinson C.V. (2008). Acetylation of L12 increases interactions in the Escherichia coli ribosomal stalk complex. J. Mol. Biol..

[105] Chang F.N. (1978). Temperature-dependent variation in the extent of methylation of ribosomal proteins L7 and L12 in Escherichia coli. J. Bacteriol..

[106] Polevoda B., Sherman F. (2007). Methylation of proteins involved in translation. Mol. Microbiol..

[107] Feid S.C., Walukiewicz H.E., Wang X., Nakayasu E.S., Rao C.V., Wolfe A.J. (2022). Regulation of translation by lysine acetylation in Escherichia coli. mBio.

[108] Soung G.Y., Miller J.L., Koc H., Koc E.C. (2009). Comprehensive analysis of phosphorylated proteins of Escherichia coli ribosomes. J. Proteome Res..

[109] Vila-Sanjurjo A. (2008). Modification of the ribosome and the translational machinery during reduced growth due to environmental stress. EcoSal Plus.

[110] Macek B., Gnad F., Soufi B., Kumar C., Olsen J.V., Mijakovic I. (2008). Phosphoproteome analysis of E. coli reveals evolutionary conservation of bacterial Ser/Thr/Tyr phosphorylation. Mol. Cell. Proteomics.

[111] Yutin N., Puigbò P., Koonin E.V., Wolf Y.I. (2012). Phylogenomics of prokaryotic ribosomal proteins. PLoS One.

[112] Lilleorg S., Reier K., Pulk A., Liiv A., Tammsalu T., Peil L. (2019). Bacterial ribosome heterogeneity: changes in ribosomal protein composition during transition into stationary growth phase. Biochimie.

[113] Lyu Z., Wilson C., Ling J. (2023). Translational fidelity during bacterial stresses and host interactions. Pathogens.

[114] Ling J., Soll D. (2010). Severe oxidative stress induces mistranslation through impairemnt of an aminoacyl-tRNA synthetase editing site. Proc. Natl. Acad. Sci. U. S. A..

[115] Ruan B., Palioura S., Sabina J., Marvin-Guy L., Kochhar S., Larossa R.A. (2008). Quality control despite mistranslation caused by an ambiguous genetic code. Proc. Natl. Acad. Sci. U. S. A..

[116] Fredriksson A., Ballesteros M., Peterson C.N., Persson O., Silhavy T.J., Nyström T. (2007). Decline in ribosomal fidelity contributes to the accumulation and stabilization of the master stress response regulator sigmaS upon carbon starvation. Genes Dev..

[117] Tamarit J., Cabiscol E., Ros J. (1998). Identification of the major oxidatively damaged proteins in Escherichia coli cells exposed to oxidative stress. J. Biol. Chem..

[118] Nagano T., Yutthanasirikul R., Hihara Y., Hisabori T., Kanamori T., Takeuchi N. (2015). Oxidation of translation factor EF-G transiently retards the translational elongation cycle in Escherichia coli. J. Biochem..

[119] Alfano M., Rizzi C., Corti D., Adduce L., Poli G. (2005). Bacterial toxins: potential weapons against HIV infection. Curr. Pharm. Des..

[120] Steinberg R., Koch H.G. (2021). The largely unexplored biology of small proteins in pro- and eukaryotes. FEBS J..

[121] Wurtmann E.J., Wolin S.L. (2009). RNA under attack: cellular handling of RNA damage. Crit. Rev. Biochem. Mol. Biol..

[122] Mohler K., Ibba M. (2017). Translational fidelity and mistranslation in the cellular response to stress. Nat. Microbiol..

[123] Pulk A., Liiv A., Peil L., Maiväli U., Nierhaus K., Remme J. (2010). Ribosome reactivation by replacement of damaged proteins. Mol. Microbiol..

[124] Tian Y., Zeng F., Raybarman A., Fatma S., Carruthers A., Li Q. (2022). Sequential rescue and repair of stalled and damaged ribosome by bacterial PrfH and RtcB. Proc. Natl. Acad. Sci. U. S. A..

[125] Wick L.M., Egli T. (2004). Molecular components of physiological stress responses in Escherichia coli. Adv. Biochem. Eng. Biotechnol..

[126] Liponska A., Yap M.F. (2021). Hibernation-promoting factor sequesters Staphylococcus aureus ribosomes to antagonize RNase R-mediated nucleolytic degradation. mBio.

[127] Prossliner T., Gerdes K., Sorensen M.A., Winther K.S. (2021). Hibernation factors directly block ribonucleases from entering the ribosome in response to starvation. Nucleic Acids Res..

[128] McKay S.L., Portnoy D.A. (2015). Ribosome hibernation facilitates tolerance of stationary-phase bacteria to aminoglycosides. Antimicrob. Agents Chemother..

[129] Polikanov Y.S., Blaha G.M., Steitz T.A. (2012). How hibernation factors RMF, HPF, and YfiA turn off protein synthesis. Science.

[130] Yoshida H., Shimada T., Ishihama A. (2018). Coordinated hibernation of transcriptional and translational apparatus during growth transition of Escherichia coli to stationary phase. mSystems.

[131] Tumer N.E., Li X.P. (2012). Interaction of ricin and Shiga toxins with ribosomes. Curr. Top. Microbiol. Immunol..

[132] Grela P., Szajwaj M., Horbowicz-Drozdzal P., Tchorzewski M. (2019). How ricin damages the ribosome. Toxins.

[133] Sandvig K., van Deurs B. (1999). Endocytosis and intracellular transport of ricin: recent discoveries. FEBS Lett..

[134] Wahome P.G., Ahlawat S., Mantis N.J. (2012). Identification of small molecules that suppress ricin-induced stress-activated signaling pathways. PLoS One.

[135] Graf M., Wilson D.N. (2019). Intracellular antimicrobial peptides targeting the protein synthesis machinery. Adv. Exp. Med. Biol..

[136] Rustgi S., Pollmann S., Buhr F., Springer A., Reinbothe C., von Wettstein D. (2014). JIP60-mediated, jasmonate- and senescence-induced molecular switch in translation toward stress and defense protein synthesis. Proc. Natl. Acad. Sci. U. S. A..

[137] Yamagishi M., Matsushima H., Wada A., Sakagami M., Fujita N., Ishihama A. (1993). Regulation of the Escherichia coli rmf gene encoding the ribosome modulation factor: growth phase- and growth rate-dependent control. EMBO J..

[138] Yoshida H., Maki Y., Kato H., Fujisawa H., Izutsu K., Wada C. (2002). The ribosome modulation factor (RMF) binding site on the 100S ribosome of Escherichia coli. J. Biochem..

[139] Yoshida H., Ueta M., Maki Y., Sakai A., Wada A. (2009). Activities of Escherichia coli ribosomes in IF3 and RMF change to prepare 100S ribosome formation on entering the stationary growth phase. Genes Cells.

[140] Yoshida H., Yamamoto H., Uchiumi T., Wada A. (2004). RMF inactivates ribosomes by covering the peptidyl transferase centre and entrance of peptide exit tunnel. Genes Cells.

[141] Wada A., Yamazaki Y., Fujita N., Ishihama A. (1990). Structure and probable genetic location of a “ribosome modulation factor” associated with 100S ribosomes in stationary-phase Escherichia coli cells. Proc. Natl. Acad. Sci. U. S. A..

[142] Ueta M., Wada C., Daifuku T., Sako Y., Bessho Y., Kitamura A. (2013). Conservation of two distinct types of 100S ribosome in bacteria. Genes Cells.

[143] Wada A. (1998). Growth phase coupled modulation of Escherichia coli ribosomes. Genes Cells.

[144] Beckert B., Turk M., Czech A., Berninghausen O., Beckmann R., Ignatova Z. (2018). Structure of a hibernating 100S ribosome reveals an inactive conformation of the ribosomal protein S1. Nat. Microbiol..

[145] Wada A., Igarashi K., Yoshimura S., Aimoto S., Ishihama A. (1995). Ribosome modulation factor: stationary growth phase-specific inhibitor of ribosome functions from Escherichia coli. Biochem. Biophys. Res. Commun..

[146] Shimada T., Yoshida H., Ishihama A. (2013). Involvement of cyclic AMP receptor protein in regulation of the rmf gene encoding the ribosome modulation factor in Escherichia coli. J. Bacteriol..

[147] DeLisa M.P., Wu C.F., Wang L., Valdes J.J., Bentley W.E. (2001). DNA microarray-based identification of genes controlled by autoinducer 2-stimulated quorum sensing in Escherichia coli. J. Bacteriol..

[148] Agafonov D.E., Kolb V.A., Spirin A.S. (2001). Ribosome-associated protein that inhibits translation at the aminoacyl-tRNA binding stage. EMBO Rep..

[149] Agafonov D.E., Spirin A.S. (2004). The ribosome-associated inhibitor A reduces translation errors. Biochem. Biophys. Res. Commun..

[150] Salmon K., Hung S.P., Mekjian K., Baldi P., Hatfield G.W., Gunsalus R.P. (2003). Global gene expression profiling in Escherichia coli K12. The effects of oxygen availability and FNR. J. Biol. Chem..

[151] Kiley P.J., Beinert H. (1998). Oxygen sensing by the global regulator, FNR: the role of the iron-sulfur cluster. FEMS Microbiol. Rev..

[152] Vila-Sanjurjo A., Schuwirth B.S., Hau C.W., Cate J.H. (2004). Structural basis for the control of translation initiation during stress. Nat. Struct. Mol. Biol..

[153] Ueta M., Yoshida H., Wada C., Baba T., Mori H., Wada A. (2005). Ribosome binding proteins YhbH and YfiA have opposite functions during 100S formation in the stationary phase of Escherichia coli. Genes Cells.

[154] Matzov D., Aibara S., Basu A., Zimmerman E., Bashan A., Yap M.F. (2017). The cryo-EM structure of hibernating 100S ribosome dimer from pathogenic Staphylococcus aureus. Nat. Commun..

[155] Yoshida H., Maki Y., Furuike S., Sakai A., Ueta M., Wada A. (2012). YqjD is an inner membrane protein associated with stationary-phase ribosomes in Escherichia coli. J. Bacteriol..

[156] Harms A., Maisonneuve E., Gerdes K. (2016). Mechanisms of bacterial persistence during stress and antibiotic exposure. Science.

[157] Aiso T., Yoshida H., Wada A., Ohki R. (2005). Modulation of mRNA stability participates in stationary-phase-specific expression of ribosome modulation factor. J. Bacteriol..

[158] Song S., Wood T.K. (2020). ppGpp ribosome dimerization model for bacterial persister formation and resuscitation. Biochem. Biophys. Res. Commun..

[159] Izutsu K., Wada C., Komine Y., Sako T., Ueguchi C., Nakura S. (2001). Escherichia coli ribosome-associated protein SRA, whose copy number increases during stationary phase. J. Bacteriol..

[160] Lacour S., Landini P. (2004). SigmaS-dependent gene expression at the onset of stationary phase in Escherichia coli: function of sigmaS-dependent genes and identification of their promoter sequences. J. Bacteriol..

[161] Semanjski M., Gratani F.L., Englert T., Nashier P., Beke V., Nalpas N. (2021). Proteome dynamics during antibiotic persistence and resuscitation. mSystems.

[162] Bubunenko M., Baker T., Court D.L. (2007). Essentiality of ribosomal and transcription antitermination proteins analyzed by systematic gene replacement in Escherichia coli. J. Bacteriol..

[163] Hauser R., Pech M., Kijek J., Yamamoto H., Titz B., Naeve F. (2012). RsfA (YbeB) proteins are conserved ribosomal silencing factors. PLoS Genet..

[164] Li X., Sun Q., Jiang C., Yang K., Hung L.W., Zhang J. (2015). Structure of ribosomal silencing factor bound to Mycobacterium tuberculosis ribosome. Structure.

[165] Nikolay R., Hilal T., Schmidt S., Qin B., Schwefel D., Vieira-Vieira C.H. (2021). Snapshots of native pre-50S ribosomes reveal a biogenesis factor network and evolutionary specialization. Mol. Cell.

[166] Khusainov I., Fatkhullin B., Pellegrino S., Bikmullin A., Liu W.T., Gabdulkhakov A. (2020). Mechanism of ribosome shutdown by RsfS in Staphylococcus aureus revealed by integrative structural biology approach. Nat. Commun..

[167] Borgese N., Righi M. (2010). Remote origins of tail-anchored proteins. Traffic.

[168] Szoke T., Nussbaum-Shochat A., Amster-Choder O. (2021). Evolutionarily conserved mechanism for membrane recognition from bacteria to mitochondria. FEBS Lett..

[169] Guo Y., Li Y., Zhan W., Wood T.K., Wang X. (2019). Resistance to oxidative stress by inner membrane protein ElaB is regulated by OxyR and RpoS. Microb. Biotechnol..

[170] Dai X., Zhu M., Warren M., Balakrishnan R., Patsalo V., Okano H. (2016). Reduction of translating ribosomes enables Escherichia coli to maintain elongation rates during slow growth. Nat. Microbiol..

[171] Reier K., Liiv A., Remme J. (2023). Ribosome protein composition mediates translation during the Escherichia coli stationary phase. Int. J. Mol. Sci..

[172] Orban K., Finkel S.E. (2022). Dps is a universally conserved dual-action DNA-binding and ferritin protein. J. Bacteriol..

[173] Leesch F., Lorenzo-Orts L., Pribitzer C., Grishkovskaya I., Roehsner J., Chugunova A. (2023). A molecular network of conserved factors keeps ribosomes dormant in the egg. Nature.

[174] Arenz S., Ramu H., Gupta P., Berninghausen O., Beckmann R., Vazquez-Laslop N. (2014). Molecular basis for erythromycin-dependent ribosome stalling during translation of the ErmBL leader peptide. Nat. Commun..

[175] Wall E., Majdalani N., Gottesman S. (2018). The complex Rcs regulatory cascade. Annu. Rev. Microbiol..

[176] Huber D., Bukau B. (2008). DegP: a protein “death star”. Structure.

[177] Troman L., Collinson I. (2021). Pushing the envelope: the mysterious journey through the bacterial secretory machinery, and beyond. Front. Microbiol..

[178] Kim H., Wu K., Lee C. (2021). Stress-responsive periplasmic chaperones in bacteria. Front. Mol. Biosci..

[179] Flores-Kim J., Darwin A.J. (2016). The phage shock protein response. Annu. Rev. Microbiol..

[180] Steinberg R., Knupffer L., Origi A., Asti R., Koch H.G. (2018). Co-translational protein targeting in bacteria. FEMS Microbiol. Lett..

[181] Park E., Rapoport T.A. (2012). Mechanisms of Sec61/SecY-mediated protein translocation across membranes. Annu. Rev. Biophys..

[182] Corey R.A., Allen W.J., Collinson I. (2016). Protein translocation: what's the problem?. Biochem. Soc. Trans..

[183] De Geyter J., Smets D., Karamanou S., Economou A. (2019). Inner membrane translocases and insertases. Subcell. Biochem..

[184] Dalbey R., Koch H.G., Kuhn A. (2017). Targeting and insertion of membrane proteins. EcoSal Plus.

[185] Van den Berg B., Clemons W.M., Collinson I., Modis Y., Hartmann E., Harrison S.C. (2004). X-ray structure of a protein-conducting channel. Nature.

[186] Carlson M.L., Stacey R.G., Young J.W., Wason I.S., Zhao Z., Rattray D.G. (2019). Profiling the E. coli membrane interactome captured in peptidisc libraries. Elife.

[187] Kedrov A., Wickles S., Crevenna A.H., van der Sluis E.O., Buschauer R., Berninghausen O. (2016). Structural dynamics of the YidC:ribosome complex during membrane protein biogenesis. Cell Rep..

[188] Bischoff L., Wickles S., Berninghaus O., van der Sluis E.O., Beckmann R. (2014). Visualization of a polytopic membrane protein during SecY-mediated membrane insertion. Nat. Commun..

[189] Smalinskaitė L., Kim M.K., Lewis A.J.O., Keenan R.J., Hegde R.S. (2022). Mechanism of an intramembrane chaperone for multipass membrane proteins. Nature.

[190] Sundaram A., Yamsek M., Zhong F., Hooda Y., Hegde R.S., Keenan R.J. (2022). Substrate-driven assembly of a translocon for multipass membrane proteins. Nature.

[191] Petriman N.A., Jauss B., Hufnagel A., Franz L., Sachelaru I., Drepper F. (2018). The interaction network of the YidC insertase with the SecYEG translocon, SRP and the SRP receptor FtsY. Sci. Rep..

[192] Welte T., Kudva R., Kuhn P., Sturm L., Braig D., Muller M. (2012). Promiscuous targeting of polytopic membrane proteins to SecYEG or YidC by the Escherichia coli signal recognition particle. Mol. Biol. Cell.

[193] Samuelson J.C., Chen M., Jiang F., Moller I., Wiedmann M., Kuhn A. (2000). YidC mediates membrane protein insertion in bacteria. Nature.

[194] Jauss B., Petriman N.A., Drepper F., Franz L., Sachelaru I., Welte T. (2019). Noncompetitive binding of PpiD and YidC to the SecYEG translocon expands the global view on the SecYEG interactome in Escherichia coli. J. Biol. Chem..

[195] Sachelaru I., Petriman N.A., Kudva R., Kuhn P., Welte T., Knapp B. (2015). YidC occupies the lateral gate of the SecYEG translocon and is sequentially displaced by a nascent membrane protein. J. Biol. Chem..

[196] Sachelaru I., Winter L., Knyazev D.G., Zimmermann M., Vogt A., Kuttner R. (2017). YidC and SecYEG form a heterotetrameric protein translocation channel. Sci. Rep..

[197] Urbanus M.L., Scotti P.A., Froderberg L., Saaf A., de Gier J.W., Brunner J. (2001). Sec-dependent membrane protein insertion: sequential interaction of nascent FtsQ with SecY and YidC. EMBO Rep..

[198] du Plessis D.J., Nouwen N., Driessen A.J. (2006). Subunit a of cytochrome o oxidase requires both YidC and SecYEG for membrane insertion. J. Biol. Chem..

[199] Celebi N., Yi L., Facey S.J., Kuhn A., Dalbey R.E. (2006). Membrane biogenesis of subunit II of cytochrome bo oxidase: contrasting requirements for insertion of N-terminal and C-terminal domains. J. Mol. Biol..

[200] McDowell M.A., Heimes M., Sinning I. (2021). Structural and molecular mechanisms for membrane protein biogenesis by the Oxa1 superfamily. Nat. Struct. Mol. Biol..

[201] Anghel S.A., McGilvray P.T., Hegde R.S., Keenan R.J. (2017). Identification of Oxa1 homologs operating in the eukaryotic endoplasmic reticulum. Cell Rep..

[202] Lewis A.J.O., Hegde R.S. (2021). A unified evolutionary origin for the ubiquitous protein transporters SecY and YidC. BMC Biol..

[203] Vögtle F.N., Koch H.G., Meisinger C. (2022). A common evolutionary origin reveals fundamental principles of protein insertases. PLoS Biol..

[204] Gu S.Q., Peske F., Wieden H.J., Rodnina M.V., Wintermeyer W. (2003). The signal recognition particle binds to protein L23 at the peptide exit of the Escherichia coli ribosome. RNA.

[205] Wang S., Jomaa A., Jaskolowski M., Yang C.I., Ban N., Shan S.O. (2019). The molecular mechanism of cotranslational membrane protein recognition and targeting by SecA. Nat. Struct. Mol. Biol..

[206] Knupffer L., Fehrenbach C., Denks K., Erichsen V., Petriman N.A., Koch H.G. (2019). Molecular mimicry of SecA and signal recognition particle binding to the bacterial ribosome. mBio.

[207] Huber D., Rajagopalan N., Preissler S., Rocco M.A., Merz F., Kramer G. (2011). SecA interacts with ribosomes in order to facilitate posttranslational translocation in bacteria. Mol. Cell.

[208] Akopian D., Shen K., Zhang X., Shan S.O. (2013). Signal recognition particle: an essential protein-targeting machine. Annu. Rev. Biochem..

[209] Ataide S.F., Schmitz N., Shen K., Ke A., Shan S.O., Doudna J.A. (2011). The crystal structure of the signal recognition particle in complex with its receptor. Science.

[210] Bernstein H.D., Poritz M.A., Strub K., Hoben P.J., Brenner S., Walter P. (1989). Model for signal sequence recognition from amino-acid sequence of 54K subunit of signal recognition particle. Nature.

[211] Braig D., Mircheva M., Sachelaru I., van der Sluis E.O., Sturm L., Beckmann R. (2011). Signal sequence-independent SRP-SR complex formation at the membrane suggests an alternative targeting pathway within the SRP cycle. Mol. Biol. Cell.

[212] Mircheva M., Boy D., Weiche B., Hucke F., Graumann P., Koch H.G. (2009). Predominant membrane localization is an essential feature of the bacterial signal recognition particle receptor. BMC Biol..

[213] Karniel A., Mrusek D., Steinchen W., Dym O., Bange G., Bibi E. (2018). Co-Translational folding intermediate dictates membrane targeting of the signal recognition particle receptor. J. Mol. Biol..

[214] de Leeuw E., te Kaat K., Moser C., Menestrina G., Demel R., de Kruijff B. (2000). Anionic phospholipids are involved in membrane association of FtsY and stimulate its GTPase activity. EMBO J..

[215] Kuhn P., Draycheva A., Vogt A., Petriman N.A., Sturm L., Drepper F. (2015). Ribosome binding induces repositioning of the signal recognition particle receptor on the translocon. J. Cell Biol..

[216] Kuhn P., Weiche B., Sturm L., Sommer E., Drepper F., Warscheid B. (2011). The bacterial SRP receptor, SecA and the ribosome use overlapping binding sites on the SecY translocon. Traffic.

[217] Halic M., Gartmann M., Schlenker O., Mielke T., Pool M.R., Sinning I. (2006). Signal recognition particle receptor exposes the ribosomal translocon binding site. Science.

[218] Schibich D., Gloge F., Pohner I., Bjorkholm P., Wade R.C., von Heijne G. (2016). Global profiling of SRP interaction with nascent polypeptides. Nature.

[219] Bornemann T., Jockel J., Rodnina M.V., Wintermeyer W. (2008). Signal sequence-independent membrane targeting of ribosomes containing short nascent peptides within the exit tunnel. Nat. Struct. Mol. Biol..

[220] Holtkamp W., Lee S., Bornemann T., Senyushkina T., Rodnina M.V., Wintermeyer W. (2012). Dynamic switch of the signal recognition particle from scanning to targeting. Nat. Struct. Mol. Biol..

[221] Denks K., Sliwinski N., Erichsen V., Borodkina B., Origi A., Koch H.G. (2017). The signal recognition particle contacts uL23 and scans substrate translation inside the ribosomal tunnel. Nat. Microbiol..

[222] Hemm M.R., Paul B.J., Miranda-Rios J., Zhang A., Soltanzad N., Storz G. (2010). Small stress response proteins in Escherichia coli: proteins missed by classical proteomic studies. J. Bacteriol..

[223] Steinberg R., Origi A., Natriashvili A., Sarmah P., Licheva M., Walker P.M. (2020). Posttranslational insertion of small membrane proteins by the bacterial signal recognition particle. PLoS Biol..

[224] Journet L., Cascales E. (2016). The type VI secretion system in Escherichia coli and related species. EcoSal Plus.

[225] Pross E., Soussoula L., Seitl I., Lupo D., Kuhn A. (2016). Membrane targeting and insertion of the C-tail protein SciP. J. Mol. Biol..

[226] Peschke M., Le Goff M., Koningstein G.M., Karyolaimos A., de Gier J.W., van Ulsen P. (2017). SRP, FtsY, DnaK and YidC are required for the biogenesis of the E. coli tail-anchored membrane proteins DjlC and flk. J. Mol. Biol..

[227] Unni R., Pintor K.L., Diepold A., Unterweger D. (2022). Presence and absence of type VI secretion systems in bacteria. Microbiology (Reading).

[228] Gupta R., Toptygin D., Kaiser C.M. (2020). The SecA motor generates mechanical force during protein translocation. Nat. Commun..

[229] Origi A., Natriashivili A., Knupffer L., Fehrenbach C., Denks K., Asti R. (2019). Yet another job for the bacterial ribosome. Microb. Cell.

[230] Castanie-Cornet M.P., Bruel N., Genevaux P. (2013). Chaperone networking facilitates protein targeting to the bacterial cytoplasmic membrane. Biochim. Biophys. Acta.

[231] Gouridis G., Karamanou S., Gelis I., Kalodimos C.G., Economou A. (2009). Signal peptides are allosteric activators of the protein translocase. Nature.

[232] Seinen A.B., Spakman D., van Oijen A.M., Driessen A.J.M. (2021). Cellular dynamics of the SecA ATPase at the single molecule level. Sci. Rep..

[233] Lill R., Dowhan W., Wickner W. (1990). The ATPase activity of SecA is regulated by acidic phospholipids, SecY nd the leader and mature domain of precursor proteins. Cell.

[234] Koch S., de Wit J.G., Vos I., Birkner J.P., Gordiichuk P., Herrmann A. (2016). Lipids activate SecA for high affinity binding to the SecYEG complex. J. Biol. Chem..

[235] Koch S., Exterkate M., López C.A., Patro M., Marrink S.J., Driessen A.J.M. (2019). Two distinct anionic phospholipid-dependent events involved in SecA-mediated protein translocation. Biochim. Biophys. Acta Biomembr..

[236] Catipovic M.A., Bauer B.W., Loparo J.J., Rapoport T.A. (2019). Protein translocation by the SecA ATPase occurs by a power-stroke mechanism. EMBO J..

[237] Allen W.J., Watkins D.W., Dillingham M.S., Collinson I. (2020). Refined measurement of SecA-driven protein secretion reveals that translocation is indirectly coupled to ATP turnover. Proc. Natl. Acad. Sci. U. S. A..

[238] Smets D., Loos M.S., Karamanou S., Economou A. (2019). Protein transport across the bacterial plasma membrane by the Sec pathway. Protein J..

[239] Zhu Z., Wang S., Shan S.O. (2022). Ribosome profiling reveals multiple roles of SecA in cotranslational protein export. Nat. Commun..

[240] Deitermann S., Sprie G.S., Koch H.G. (2005). A dual function for SecA in the assembly of single spanning membrane proteins in Escherichia coli. J. Biol. Chem..

[241] Neumann-Haefelin C., Schafer U., Muller M., Koch H.G. (2000). SRP-dependent co-translational targeting and SecA-dependent translocation analyzed as individual steps in the export of a bacterial protein. EMBO J..

[242] Koch H.G., Moser M., Muller M. (2003). Signal recognition particle-dependent protein targeting, universal to all kingdoms of life. Rev. Physiol. Biochem. Pharmacol..

[243] Wang B., Dai P., Ding D., Del Rosario A., Grant R.A., Pentelute B.L. (2019). Affinity-based capture and identification of protein effectors of the growth regulator ppGpp. Nat. Chem. Biol..

[244] Draycheva A., Lee S., Wintermeyer W. (2018). Cotranslational protein targeting to the membrane: nascent-chain transfer in a quaternary complex formed at the translocon. Sci. Rep..

[245] Egea P.F., Shan S.O., Napetschnig J., Savage D.F., Walter P., Stroud R.M. (2004). Substrate twinning activates the signal recognition particle and its receptor. Nature.

[246] Jomaa A., Fu Y.H., Boehringer D., Leibundgut M., Shan S.O., Ban N. (2017). Structure of the quaternary complex between SRP, SR, and translocon bound to the translating ribosome. Nat. Commun..

[247] Schuster S., Vavra M., Greim L., Kern W.V. (2021). Exploring the contribution of the AcrB homolog MdtF to drug resistance and dye efflux in a multidrug resistant E. coli isolate. Antibiotics (Basel).

[248] Vijayakumar S.R., Kirchhof M.G., Patten C.L., Schellhorn H.E. (2004). RpoS-regulated genes of Escherichia coli identified by random lacZ fusion mutagenesis. J. Bacteriol..

[249] Sitnikov D.M., Schineller J.B., Baldwin T.O. (1996). Control of cell division in Escherichia coli: regulation of transcription of ftsQA involves both rpoS and SdiA-mediated autoinduction. Proc. Natl. Acad. Sci. U. S. A..

[250] Ulbrandt N.D., Newitt J.A., Bernstein H.D. (1997). The E. coli signal recognition particle is required for the insertion of a subset of inner membrane proteins. Cell.

[251] Yosef I., Bochkareva E.S., Adler J., Bibi E. (2010). Membrane protein biogenesis in Ffh- or FtsY-depleted Escherichia coli. PLoS One.

[252] Koch H.G., Hengelage T., Neumann-Haefelin C., MacFarlane J., Hoffschulte H.K., Schimz K.L. (1999). *In vitro* studies with purified components reveal signal recognition particle (SRP) and SecA/SecB as constituents of two independent protein-targeting pathways of Escherichia coli. Mol. Biol. Cell.

[253] Butkus M.E., Prundeanu L.B., Oliver D.B. (2003). Translocon “pulling” of nascent SecM controls the duration of its translational pause and secretion-responsive secA regulation. J. Bacteriol..

[254] Murakami A., Nakatogawa H., Ito K. (2004). Translation arrest of SecM is essential for the basal and regulated expression of SecA. Proc. Natl. Acad. Sci. U. S. A..

[255] Rawat S., Zhu L., Lindner E., Dalbey R.E., White S.H. (2015). SecA drives transmembrane insertion of RodZ, an unusual single-span membrane protein. J. Mol. Biol..

[256] Lindner E., White S.H. (2019). Dropping out and other fates of transmembrane segments inserted by the SecA ATPase. J. Mol. Biol..

[257] Koch H.G., Muller M. (2000). Dissecting the translocase and integrase functions of the Escherichia coli SecYEG translocon. J. Cell Biol..

[258] Kannaiah S., Amster-Choder O. (2014). Protein targeting via mRNA in bacteria. Biochim. Biophys. Acta.

[259] Kannaiah S., Livny J., Amster-Choder O. (2019). Spatiotemporal organization of the E. coli transcriptome: translation independence and engagement in regulation. Mol. Cell.

[260] Nevo-Dinur K., Nussbaum-Shochat A., Ben-Yehuda S., Amster-Choder O. (2011). Translation-independent localization of mRNA in E. coli. Science.

[261] Sarmah P., Shang W., Origi A., Licheva M., Kraft C., Ulbrich M. (2023). mRNA targeting eliminates the need for the signal recognition particle during membrane protein insertion in bacteria. Cell Rep..

[262] Jagannathan S., Hsu J.C., Reid D.W., Chen Q., Thompson W.J., Moseley A.M. (2014). Multifunctional roles for the protein translocation machinery in RNA anchoring to the endoplasmic reticulum. J. Biol. Chem..

[263] Pyhtila B., Zheng T., Lager P.J., Keene J.D., Reedy M.C., Nicchitta C.V. (2008). Signal sequence- and translation-independent mRNA localization to the endoplasmic reticulum. RNA.

[264] Frauenfeld J., Gumbart J., Sluis E.O., Funes S., Gartmann M., Beatrix B. (2011). Cryo-EM structure of the ribosome-SecYE complex in the membrane environment. Nat. Struct. Mol. Biol..

[265] Prinz A., Behrens C., Rapoport T.A., Hartmann E., Kalies K.U. (2000). Evolutionarily conserved binding of ribosomes to the translocation channel via the large ribosomal RNA. EMBO J..

[266] Cheng Z., Jiang Y., Mandon E.C., Gilmore R. (2005). Identification of cytoplasmic residues of Sec61p involved in ribosome binding and cotranslational translocation. J. Cell Biol..

[267] Izutsu K., Wada A., Wada C. (2001). Expression of ribosome modulation factor (RMF) in Escherichia coli requires ppGpp. Genes Cells.

[268] Zhao L., Cui Y., Fu G., Xu Z., Liao X., Zhang D. (2021). Signal recognition particle suppressor screening reveals the regulation of membrane protein targeting by the translation rate. mBio.

[269] Zhao L., Fu G., Cui Y., Xu Z., Cai T., Zhang D. (2021). Compensating complete loss of signal recognition particle during co-translational protein targeting by the translation speed and accuracy. Front. Microbiol..

[270] Xie L., Jakob U. (2019). Inorganic polyphosphate, a multifunctional polyanionic protein scaffold. J. Biol. Chem..

[271] Ahmad S., Wang B., Walker M.D., Tran H.R., Stogios P.J., Savchenko A. (2019). An interbacterial toxin inhibits target cell growth by synthesizing (p)ppApp. Nature.

[272] Bruhn-Olszewska B., Molodtsov V., Sobala M., Dylewski M., Murakami K.S., Cashel M. (2018). Structure-function comparisons of (p)ppApp vs (p)ppGpp for Escherichia coli RNA polymerase binding sites and for rrnB P1 promoter regulatory responses *in vitro*. Biochim. Biophys. Acta Gene Regul. Mech..

[273] Potrykus K., Thomas N.E., Bruhn-Olszewska B., Sobala M., Dylewski M., James T. (2020). Estimates of rel(seq), Mesh1, and SAH(mex) hydrolysis of (p)ppGpp and (p)ppApp by thin layer chromatography and NADP/NADH coupled assays. Front. Microbiol..

[274] Haas T.M., Laventie B.J., Lagies S., Harter C., Prucker I., Ritz D. (2022). Photoaffinity capture compounds to profile the magic spot nucleotide interactomes. Angew. Chem. Int. Ed. Engl..

[275] Figueira R., Brown D.R., Ferreira D., Eldridge M.J., Burchell L., Pan Z. (2015). Adaptation to sustained nitrogen starvation by Escherichia coli requires the eukaryote-like serine/threonine kinase YeaG. Sci. Rep..

[276] White S.H., von Heijne G. (2008). How translocons select transmembrane helices. Annu. Rev. Biophys..

[277] Knyazev D.G., Kuttner R., Zimmermann M., Sobakinskaya E., Pohl P. (2018). Driving forces of translocation through bacterial translocon SecYEG. J. Membr. Biol..

[278] Alami M., Luke I., Deitermann S., Eisner G., Koch H.G., Brunner J. (2003). Differential interactions between a twin-arginine signal peptide and its translocase in Escherichia coli. Mol. Cell.

[279] Stolle P., Hou B., Brüser T. (2016). The Tat substrate CueO is transported in an incomplete folding state. J. Biol. Chem..

[280] Öztürk Y., Blaby-Haas C.E., Daum N., Andrei A., Rauch J., Daldal F. (2021). Maturation of rhodobacter capsulatus multicopper oxidase CutO depends on the CopA copper efflux pathway and requires the cutF product. Front. Microbiol..

[281] Ricci D.P., Silhavy T.J. (2019). Outer membrane protein insertion by the β-barrel assembly machine. EcoSal Plus.

[282] Konovalova A., Kahne D.E., Silhavy T.J. (2017). Outer membrane biogenesis. Annu. Rev. Microbiol..

[283] Bos M.P., Robert V., Tommassen J. (2007). Biogenesis of the gram-negative bacterial outer membrane. Annu. Rev. Microbiol..

[284] Lemke J.J., Sanchez-Vazquez P., Burgos H.L., Hedberg G., Ross W., Gourse R.L. (2011). Direct regulation of Escherichia coli ribosomal protein promoters by the transcription factors ppGpp and DksA. Proc. Natl. Acad. Sci. U. S. A..

[285] Coenye T., Vandamme P. (2005). Organisation of the S10, spc and alpha ribosomal protein gene clusters in prokaryotic genomes. FEMS Microbiol. Lett..

[286] Ikegami A., Nishiyama K., Matsuyama S., Tokuda H. (2005). Disruption of rpmJ encoding ribosomal protein L36 decreases the expression of secY upstream of the spc operon and inhibits protein translocation in Escherichia coli. Biosci. Biotechnol. Biochem..

[287] Downing W.L., Sullivan S.L., Gottesman M.E., Dennis P.P. (1990). Sequence and transcriptional pattern of the essential Escherichia coli secE-nusG operon. J. Bacteriol..

[288] Nishiyama K., Tokuda H. (2005). Genes coding for SecG and Leu2-tRNA form an operon to give an unusual RNA comprising mRNA and a tRNA precursor. Biochim. Biophys. Acta.

[289] Prossliner T., Agrawal S., Heidemann D.F., Sørensen M.A., Svenningsen S.L. (2023). tRNAs are stable after all: pitfalls in quantification of tRNA from starved Escherichia coli cultures exposed by validation of RNA purification methods. mBio.

[290] Sørensen M.A., Fehler A.O., Lo Svenningsen S. (2018). Transfer RNA instability as a stress response in Escherichia coli: rapid dynamics of the tRNA pool as a function of demand. RNA Biol..

[291] Soufi B., Krug K., Harst A., Macek B. (2015). Characterization of the E. coli proteome and its modifications during growth and ethanol stress. Front. Microbiol..

[292] Soufi B., Macek B. (2015). Global analysis of bacterial membrane proteins and their modifications. Int. J. Med. Microbiol..

[293] Yu Z., Laven M., Klepsch M., de Gier J.W., Bitter W., van Ulsen P. (2011). Role for Escherichia coli YidD in membrane protein insertion. J. Bacteriol..

[294] Hansen F.G., Hansen E.B., Atlung T. (1982). The nucleotide sequence of the dnaA gene promoter and of the adjacent rpmH gene, coding for the ribosomal protein L34, of Escherichia coli. EMBO J..

[295] Traxler M.F., Summers S.M., Nguyen H.T., Zacharia V.M., Hightower G.A., Smith J.T. (2008). The global, ppGpp-mediated stringent response to amino acid starvation in Escherichia coli. Mol. Microbiol..

[296] van der Laan M., Bechtluft P., Kol S., Nouwen N., Driessen A.J. (2004). F1F0 ATP synthase subunit c is a substrate of the novel YidC pathway for membrane protein biogenesis. J. Cell Biol..

[297] Yi L., Jiang F., Chen M., Cain B., Bolhuis A., Dalbey R.E. (2003). YidC is strictly required for membrane insertion of subunits a and c of the F(1)F(0)ATP synthase and SecE of the SecYEG translocase. Biochemistry.

[298] van Bloois E., Haan G.J., de Gier J.W., Oudega B., Luirink J. (2006). Distinct requirements for translocation of the N-tail and C-tail of the Escherichia coli inner membrane protein CyoA. J. Biol. Chem..

[299] Rabbers I., Bruggeman F.J. (2022). Escherichia coli robustly expresses ATP synthase at growth rate-maximizing concentrations. FEBS J..

[300] Cotter P.A., Chepuri V., Gennis R.B., Gunsalus R.P. (1990). Cytochrome o (cyoABCDE) and d (cydAB) oxidase gene expression in Escherichia coli is regulated by oxygen, pH, and the fnr gene product. J. Bacteriol..

[301] Baptista I.S.C., Kandavalli V., Chauhan V., Bahrudeen M.N.M., Almeida B.L.B., Palma C.S.D. (2022). Sequence-dependent model of genes with dual σ factor preference. Biochim. Biophys. Acta Gene Regul. Mech..

[302] Dartigalongue C., Missiakas D., Raina S. (2001). Characterization of the Escherichia coli sigma E regulon. J. Biol. Chem..

[303] Okuda S., Tokuda H. (2011). Lipoprotein sorting in bacteria. Annu. Rev. Microbiol..

[304] Ryabichko S., Ferreira V.M., Vitrac H., Kiyamova R., Dowhan W., Bogdanov M. (2020). Cardiolipin is required *in vivo* for the stability of bacterial translocon and optimal membrane protein translocation and insertion. Sci. Rep..

[305] Collinson I. (2019). The dynamic ATP-driven mechanism of bacterial protein translocation and the critical role of phospholipids. Front. Microbiol..

[306] Corey R.A., Pyle E., Allen W.J., Watkins D.W., Casiraghi M., Miroux B. (2018). Specific cardiolipin-SecY interactions are required for proton-motive force stimulation of protein secretion. Proc. Natl. Acad. Sci. U. S. A..

[307] Prabudiansyah I., Kusters I., Caforio A., Driessen A.J. (2015). Characterization of the annular lipid shell of the Sec translocon. Biochim. Biophys. Acta.

[308] Baker L.A., Sinnige T., Schellenberger P., de Keyzer J., Siebert C.A., Driessen A.J.M. (2018). Combined (1)H-detected solid-state NMR spectroscopy and electron cryotomography to study membrane proteins across resolutions in native environments. Structure.

[309] Chen Y., Capponi S., Zhu L., Gellenbeck P., Freites J.A., White S.H. (2017). YidC insertase of Escherichia coli: water accessibility and membrane shaping. Structure.

[310] Kedrov A., Sustarsic M., de Keyzer J., Caumanns J.J., Wu Z.C., Driessen A.J. (2013). Elucidating the native architecture of the YidC: ribosome complex. J. Mol. Biol..

[311] Hao B., Zhou W., Theg S.M. (2022). Hydrophobic mismatch is a key factor in protein transport across lipid bilayer membranes via the Tat pathway. J. Biol. Chem..

[312] Rathmann C., Schlösser A.S., Schiller J., Bogdanov M., Brüser T. (2017). Tat transport in Escherichia coli requires zwitterionic phosphatidylethanolamine but no specific negatively charged phospholipid. FEBS Lett..

[313] Stockwald E.R., Steger L.M.E., Vollmer S., Gottselig C., Grage S.L., Bürck J. (2023). Length matters: functional flip of the short TatA transmembrane helix. Biophys. J..

[314] Braig D., Bar C., Thumfart J.O., Koch H.G. (2009). Two cooperating helices constitute the lipid-binding domain of the bacterial SRP receptor. J. Mol. Biol..

[315] Gold V.A., Robson A., Bao H., Romantsov T., Duong F., Collinson I. (2010). The action of cardiolipin on the bacterial translocon. Proc. Natl. Acad. Sci. U. S. A..

[316] Kamel M., Löwe M., Schott-Verdugo S., Gohlke H., Kedrov A. (2022). Unsaturated fatty acids augment protein transport via the SecA:SecYEG translocon. FEBS J..

[317] Rowlett V.W., Mallampalli V., Karlstaedt A., Dowhan W., Taegtmeyer H., Margolin W. (2017). Impact of membrane phospholipid alterations in Escherichia coli on cellular function and bacterial stress adaptation. J. Bacteriol..

[318] Marr A.G., Ingraham J.L. (1962). Effect of temperature on the composition of fatty acids in Escherichia coli. J. Bacteriol..

[319] DiRusso C.C., Nyström T. (1998). The fats of Escherichia coli during infancy and old age: regulation by global regulators, alarmones and lipid intermediates. Mol. Microbiol..

[320] Gitter B., Diefenbach R., Keweloh H., Riesenberg D. (1995). Influence of stringent and relaxed response on excretion of recombinant proteins and fatty acid composition in Escherichia coli. Appl. Microbiol. Biotechnol..

[321] Bianco C.M., Fröhlich K.S., Vanderpool C.K. (2019). Bacterial cyclopropane fatty acid synthase mRNA is targeted by activating and repressing small RNAs. J. Bacteriol..

[322] Kralj T., Nuske M., Hofferek V., Sani M.A., Lee T.H., Separovic F. (2022). Multi-omic analysis to characterize metabolic adaptation of the E. coli lipidome in response to environmental stress. Metabolites.

[323] Keller R., Ariöz C., Hansmeier N., Stenberg-Bruzell F., Burstedt M., Vikström D. (2015). The Escherichia coli envelope stress sensor CpxA responds to changes in lipid bilayer properties. Biochemistry.

[324] Borrok M.J., Zhu Y., Forest K.T., Kiessling L.L. (2009). Structure-based design of a periplasmic binding protein antagonist that prevents domain closure. ACS Chem. Biol..

[325] Raivio T.L., Silhavy T.J. (2001). Periplasmic stress and ECF sigma factors. Annu. Rev. Microbiol..

[326] Mitchell A.M., Silhavy T.J. (2019). Envelope stress responses: balancing damage repair and toxicity. Nat. Rev. Microbiol..

[327] Mayer C., Borges A., Flament-Simon S.C., Simões M. (2023). Quorum sensing architecture network in Escherichia coli virulence and pathogenesis. FEMS Microbiol. Rev..

[328] Colin R., Ni B., Laganenka L., Sourjik V. (2021). Multiple functions of flagellar motility and chemotaxis in bacterial physiology. FEMS Microbiol. Rev..

[329] Nakatogawa H., Murakami A., Ito K. (2004). Control of SecA and SecM translation by protein secretion. Curr. Opin. Microbiol..

[330] Ishii E., Chiba S., Hashimoto N., Kojima S., Homma M., Ito K. (2015). Nascent chain-monitored remodeling of the Sec machinery for salinity adaptation of marine bacteria. Proc. Natl. Acad. Sci. U. S. A..

[331] Ito K., Mori H., Chiba S. (2018). Monitoring substrate enables real-time regulation of a protein localization pathway. FEMS Microbiol. Lett..

[332] Öztürk Y., Andrei A., Blaby-Haas C.E., Daum N., Daldal F., Koch H.G. (2023). Metabolic sensing of extracytoplasmic copper availability via translational control by a nascent exported protein. mBio.

[333] Roy G., Antoine R., Schwartz A., Slupek S., Rivera-Millot A., Boudvillain M. (2022). Posttranscriptional regulation by copper with a new upstream open reading frame. mBio.

[334] Rademacher C., Moser R., Lackmann J.W., Klinkert B., Narberhaus F., Masepohl B. (2012). Transcriptional and posttranscriptional events control copper-responsive expression of a Rhodobacter capsulatus multicopper oxidase. J. Bacteriol..

[335] Lim B., Miyazaki R., Neher S., Siegele D.A., Ito K., Walter P. (2013). Heat shock transcription factor sigma32 co-opts the signal recognition particle to regulate protein homeostasis in E. coli. PLoS Biol..

[336] Sauerbrei B., Arends J., Schünemann D., Narberhaus F. (2020). Lon protease removes excess signal recognition particle protein in Escherichia coli. J. Bacteriol..

[337] Kuroda A. (2006). A polyphosphate-lon protease complex in the adaptation of Escherichia coli to amino acid starvation. Biosci. Biotechnol. Biochem..

[338] Gray M.J. (2019). Inorganic polyphosphate accumulation in Escherichia coli is regulated by DksA but not by (p)ppGpp. J. Bacteriol..

[339] Ji X., Zou J., Peng H., Stolle A.S., Xie R., Zhang H. (2019). Alarmone Ap4A is elevated by aminoglycoside antibiotics and enhances their bactericidal activity. Proc. Natl. Acad. Sci. U. S. A..

[340] Luciano D.J., Levenson-Palmer R., Belasco J.G. (2019). Stresses that raise Np(4)A levels induce protective nucleoside tetraphosphate capping of bacterial RNA. Mol. Cell.

[341] Dawan J., Ahn J. (2022). Bacterial stress responses as potential targets in overcoming antibiotic resistance. Microorganisms.

[342] Wexselblatt E., Oppenheimer-Shaanan Y., Kaspy I., London N., Schueler-Furman O., Yavin E. (2012). Relacin, a novel antibacterial agent targeting the stringent response. PLoS Pathog..

[343] de la Fuente-Núñez C., Korolik V., Bains M., Nguyen U., Breidenstein E.B., Horsman S. (2012). Inhibition of bacterial biofilm formation and swarming motility by a small synthetic cationic peptide. Antimicrob. Agents Chemother..

[344] de la Fuente-Núñez C., Reffuveille F., Haney E.F., Straus S.K., Hancock R.E. (2014). Broad-spectrum anti-biofilm peptide that targets a cellular stress response. PLoS Pathog..

[345] Dutta N.K., Klinkenberg L.G., Vazquez M.J., Segura-Carro D., Colmenarejo G., Ramon F. (2019). Inhibiting the stringent response blocks Mycobacterium tuberculosis entry into quiescence and reduces persistence. Sci. Adv..

[346] Schmidt A., Kochanowski K., Vedelaar S., Ahrné E., Volkmer B., Callipo L. (2016). The quantitative and condition-dependent Escherichia coli proteome. Nat. Biotechnol..

[347] Neidhardt F.C., Bloch P.L., Smith D.F. (1974). Culture medium for enterobacteria. J. Bacteriol..

[348] Arnold R.J., Reilly J.P. (1999). Observation of Escherichia coli ribosomal proteins and their posttranslational modifications by mass spectrometry. Anal. Biochem..

